# Tautomeric Equilibrium in 1-Benzamidoisoquinoline Derivatives

**DOI:** 10.3390/molecules28031101

**Published:** 2023-01-22

**Authors:** Patryk Rybczyński, Anna Kaczmarek-Kędziera, Alex Iglesias-Reguant, Damian Plażuk, Borys Ośmiałowski

**Affiliations:** 1 Faculty of Chemistry, Nicolaus Copernicus University in Toruń, Gagarina 7, 87100 Toruń, Poland; 2 Institute of Computational Chemistry and Catalysis and Department of Chemistry, University of Girona, Campus de Montilivi, 17003 Girona, Spain; 3 Laboratory of Molecular Spectroscopy, Department of Organic Chemistry, Faculty of Chemistry, University of Łódź, ul. Tamka 12, 91403 Łódź, Poland

**Keywords:** tautomeric equilibrium, DFT calculations, NMR spectroscopy

## Abstract

In this study, the tautomeric equilibrium of a sequence of 1-benzamidoisoquinoline derivatives was investigated with the tools of NMR spectroscopy and computational chemistry. The equilibrium between different tautomers in these systems could be controlled via the substitution effect, and the relative content of the amide form varied from 74% for the strong electron-donating NMe${}_{2}$ substituent to 38% for the strong electron-accepting NO${}_{2}$ group in the phenyl ring. In contrast to the previously investigated 2-phenacylquinoline derivatives, the most stable and thus most abundant tautomer in the 1-benzamidoisoquinoline series except the two most electron-accepting substituents was an amide. The intramolecular hydrogen bond present in the enol tautomer competed with the intermolecular hydrogen bonds created with the solvent molecules and thus was not a sufficient factor to favor this tautomer in the mixture. Although routinely computational studies of tautomeric equilibrium are performed within the continuum solvent models, it is proven here that the inclusion of the explicit solvent is mandatory in order to reproduce the experimental tendencies observed for this type of system, facilitating strong intermolecular hydrogen bonds.

## 1. Introduction

Prototropic tautomerism is the process of a proton transfer between two atoms in the same molecule. Generally, this phenomenon occurs between the X–H fragment (X = N, O, S, C) and Y possessing the lone electron pair (Y = N, O, S), and the equilibrium is established between the two different tautomeric forms occurring in the reaction mixture simultaneously. This kind of reaction is typical for β-dicarbonyl compounds, such as β-diketones, β-ketoesters, or β-enaminones (β-aminoacroleins), and in heterocyclic ketones such as 2-phenacylpiridines [1]. It is also important in biochemistry and occurs in biogenic amines (adenine and guanine) [2] and porphyrin analogs with tetrapyrrolic frameworks [3]. Deep knowledge of the tautomerism process may lead to an understanding of the mechanisms of various chemical or biochemical reactions. For example, it was proposed that double proton transfer along the hydrogen bonds of DNA can create short-lived, but biologically relevant, point mutations that can further lead to gene mutation [4,5]. Tautomerism may also affect the advanced photophysical and photochemical features of dyes. Fluorescent probes based on the phenomenon of excited-state intramolecular proton transfer (ESIPT) are gaining more and more attention. ESIPT is a photochemical process, with the ground state of fluorophores most often existing in an enol form. After excitation, the electronic structure of such molecules can be changed, resulting in greater acidity for the hydrogen bond donor group and increased basicity for the hydrogen bond acceptor. As a consequence, an extremely fast enol-to-keto phototautomerization (proton transfer in an excited state) takes place, with the excited state enol quickly converting to its excited keto form. After radiatively decaying back to the ground state, reverse proton transfer (RPT) occurs to recover the enol form [6]. However, the practical, efficient realization of ESIPT for the achievement of the bathochromic emission shift requires the thorough control of the tautomeric equilibrium of the ground state in the analyzed system as well. That was the main motivation for the current research.

The abovementioned practical importance of the tautomerism continuously established in the heterocyclic molecules has induced the basic research in this area focused on both the qualitative and quantitative determination of the tautomeric mixture composition and the strategies of controlling the ratio of tautomeric forms. This may be achieved based on either the structural modification of the system itself (substituents, benzannulation, etc.), the possible hydrogen bond formation upon conformational change [7], or on environmental factors such as temperature or solvent character [8]. The original study devoted to the benzannulated derivatives of 2-phenacylpyridine provided a clear correlation of the tautomeric mixture composition with the strength of the intramolecular hydrogen bond in β-enaminone isomers, increased by the presence of the electron-withdrawing substituents in the phenyl ring [9,10]. Thus, one could expect that the balance of tautomers, affected by the acidity of the methylene hydrogen atoms, can be also manipulated by the exchange of the methylene group in the chain by the isoelectronic −NH− fragment, leading to the amide moiety. The tautomerism in amides is not as frequent when one focuses on distinguishing both forms in solution. It should be underlined that there are only rare examples of enols in such types of systems reported in the literature [11,12], showing their importance in the synthesis [13].

**Figure 1:**
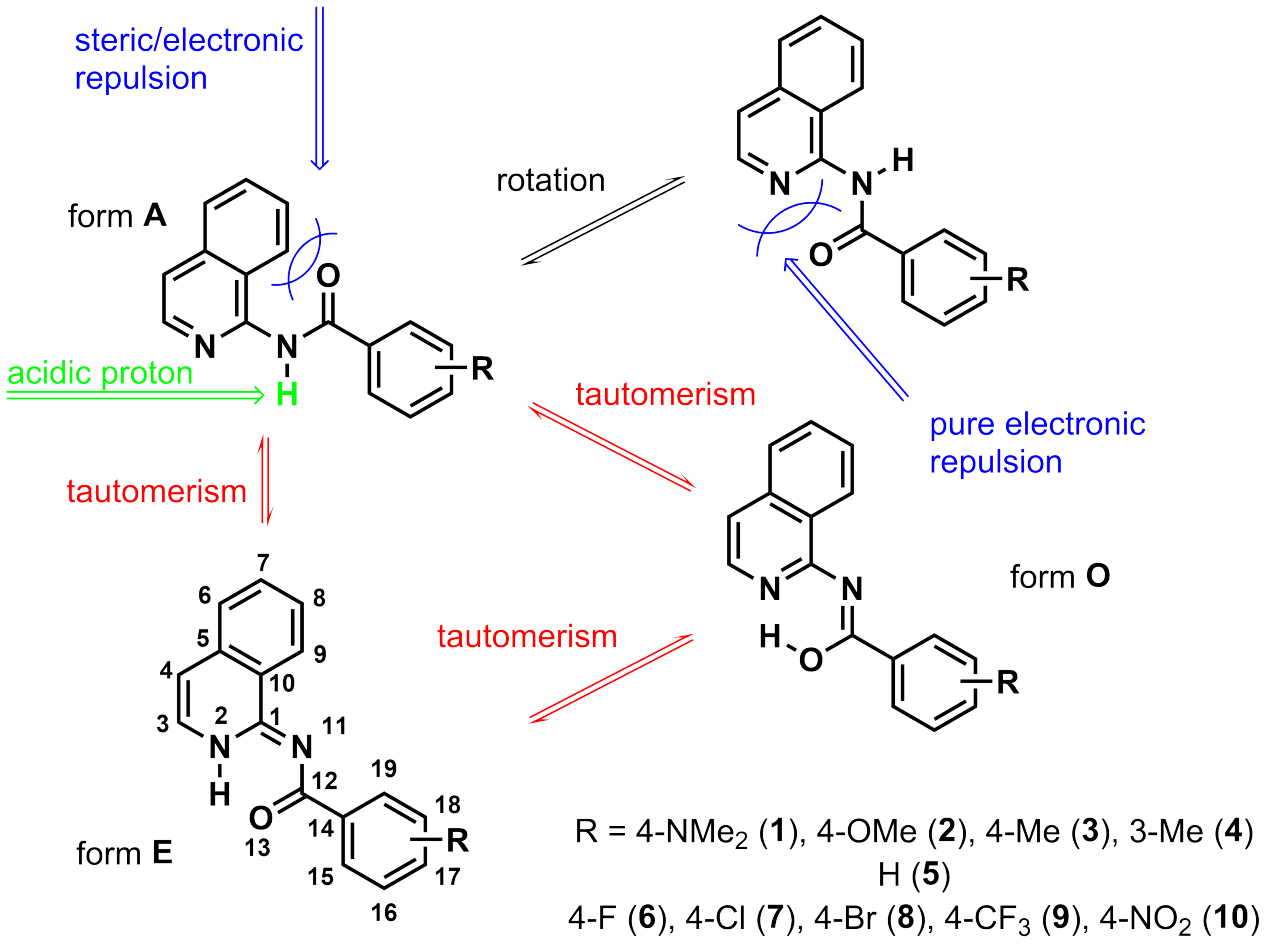
The tautomerism and steric/electronic repulsion in the studied series of molecules.

From a structural point of view, the close arrangement of the groups within the organic molecule may cause intramolecular interactions, resulting in changes in geometry or providing another path to dissipate the strain energy. In the case of amides, the −CONH− moiety is placed in a specific way (compare Figure 1): (*a*) The C=O group in ’form **A**’ can be located close to the benzo ring fused with the heterocyclic one (Figure 1), and (*b*) the same C=O group may be in close contact with the heterocyclic nitrogen atom after the rotation around the single bond (Figure 1, structure in upper right corner). Since the C=O/benzo ring interaction has a steric character (point *a*), and the C=O/N interaction (point *b*) is strongly repulsive due to the presence of electron lone pairs at both atoms, the relaxed geometry of form **A** is twisted, although in general, the conjugated molecules tend to lower their energy through planarization. Yet, the specific competition between the abovementioned features, together with the presence of the acidic proton in the structure, makes it possible to lower the energy through the proton shift between atoms.

Therefore, the aim of the present study was a detailed investigation of the tautomeric equilibrium in a series of amides, which are isoquinoline derivatives, presented schematically in Figure 1 and differing by the acidity of the labile proton obtained by the introduction of various substituents in a phenyl ring. NMR spectroscopy was applied as a precise tool to qualitatively determine the tautomeric equilibrium in heterocyclic systems. This technique provides not only information about the ratio of tautomers but also their structure, which can be further compared with the results of quantum chemical calculations (geometry, shielding, and relative energy). In order to confirm our experimental findings and justify the tautomer preferences in the systems containing a palette of substituents, quantum chemistry approaches were applied.

## 2. Results

### 2.1. Experiments

Figure 1 shows the three possible tautomers of the investigated systems: amide (**A**), enamine (**E**), and enol (**O**). In the current work, the presence of only two tautomeric forms of **1**–**10** is clearly seen in the ${}^{1}$H NMR spectra in DMSO-D${}_{6}$. The signals of both tautomeric forms appear in nuclear magnetic resonance spectroscopy only because the proton transfer between **A** and **E** is slow on the NMR time scale. Tautomeric equilibria are often of primary interest in aqueous solutions. However, due to the poor solubility of the investigated compounds, it is challenging to record NMR spectra in D${}_{2}$O. Moreover, the protons most sensitive to the change in the tautomeric state (NH) would then be substituted with deuterium, resulting in signal disappearance. Generally, tautomeric forms with intramolecular hydrogen bonds are preferred in non-polar solvents relative to water. An exception to this rule can be found for the 2-phenacylpyrazine derivative, where the form with intramolecular hydrogen bonding is beneficial both in aqueous and non-polar environments [14]. This is similar to the case of isoquinoline derivatives studied here, where the two tautomeric forms were present in the mixture despite the use of a polar aprotic solvent (DMSO), which intuitively should favor the **A** form.

**Figure 2:**
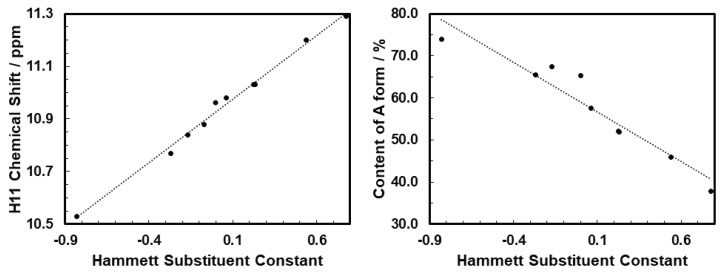
Correlation between the Hammett substituent constant and the ${}^{1}$H NMR chemical shifts for H11 **A** form (**left**, R${}^{2}$ = 0.99) and content of **A** (**right** panel, R${}^{2}$ = 0.91).

For the abovementioned reason, in the present study, dimethylsulfoxide was selected as a solvent, to determine the **A** and **E** population ratio. In the ${}^{1}$H NMR spectrum in DMSO-D${}_{6}$, the proton H2 at the heterocyclic nitrogen atom (Figure 1) of the **E** form exhibited a chemical shift close to 14.8 ppm, while for the **A** form, proton H11 in the exocyclic NH group was detected in the range from 10 to 11 ppm. This clear separation of the two signals allowed for the precise determination of the populations of different forms in the mixture through the ratio of signal integration (compare Table 1). While the integration of NH labile protons may not be exact, the CH protons were also analyzed in order to show that, in this case, the NH integration was in line with the CH ones. The amide-to-enamine form ratio decreased with the decreasing electron-donating character and further with the increasing electron-withdrawing character of the substituent R. The linear correlation between the percentage fraction of **A** form and the Hammett substituent constant is presented on the right panel of Figure 2 (R${}^{2}=0.91$). For compound **1**, carrying a strong electron-donating group, the dominating tautomer was **A**, and its population amounted to about 74%. For compound **10** with the NO${}_{2}$ group, form **E** was observed as the dominating tautomer (in the fraction equal to 62%).

This confirms that the tautomeric equilibrium can also be precisely controlled in the series of 1-benzamidoisoquinolines through the substituent effect.

Table 1 lists the chemical shifts for hydrogen atoms H2, H11 (N—H), H3, and H9 (C—H) in the isoquinoline fragment for both **A** and **E** tautomers.

The chemical shift of the H2 proton in the **E** tautomer was practically constant across the series. On the other hand, it is clear that the chemical shift for the H11 proton (form **A**) correlated linearly with the Hammett substituent constant (R${}^{2}$= 0.99; see the left panel in Figure 2), showing that the substituent effect was transmitted to that moiety. A similarly high correlation was observed for the content of the **A** tautomer.

The ${}^{1}$H NMR chemical shifts for the remaining chosen protons (H3 and H9) were also affected by a substituent. This was especially seen for the H9 proton, where the R${}^{2}$ values were 0.99 and 0.92 for **E** and **A** forms, respectively (see Figure S31). From a structural point of view, the most important observation was that for the H9 proton, a significantly different chemical shift was observed for **A** and **E** forms. This is related to the deshielding arising from the magnetic anisotropy of the carbon–oxygen bond [15,16]. The relaxed structures of **A** and **E** have a spatially different arrangement of C=O moiety. As a consequence, the chemical shift δ(H9) differed by about 0.9 ppm in both tautomers.

**Figure 3:**
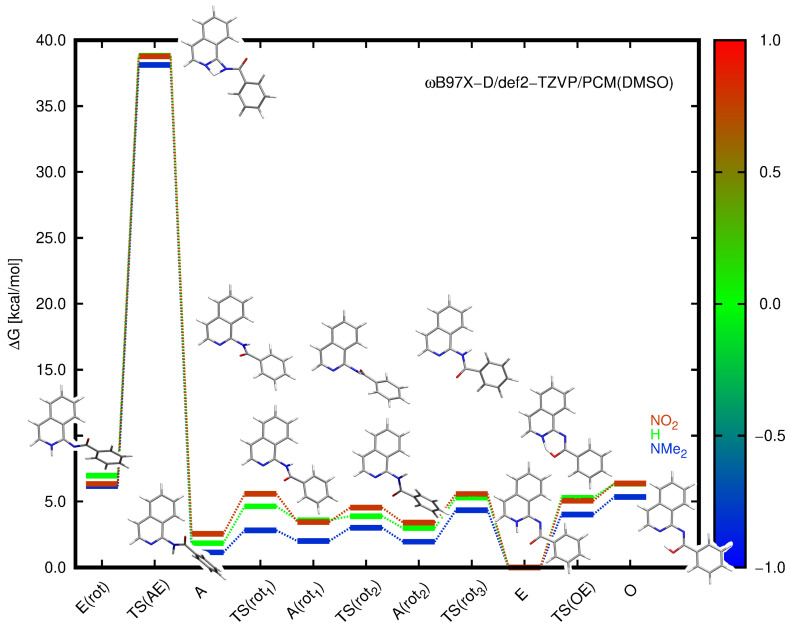
Relative Gibbs free energies (kcal/mol) for the NMe${}_{2}$, H, and NO${}_{2}$-substituted derivatives (**1**, **5**, and **10**) calculated within the ωB97X-D/def2-TZVP approach for the implicit solvent model. The color scale corresponds to the Hammett constant value of the substituent, and the presented structures are given for the unsubstituted system **5**. TS(**AE**) and TS(**OE**) denote the transition state structures for the proton transfer between the corresponding tautomers, and the remaining TS labels apply to the rotation of the amide along the single C-N bond. **E**(rot) and **A**(rot${}_{n}$) stand for the optimized rotamers of the corresponding tautomers.

Since DMSO is a very polar solvent, it was expected that the water content (from DMSO or the solute) would vary from one sample to the other. Thus, in order to investigate the effect of water content on the equilibrium in the solution, a sample of compound **6** was prepared, and the proton spectrum was recorded. A small amount of pure water (0.10 mL for 0.60 mL of DMSO) was added, again determining the ratio of both tautomers in the mixture by NMR. It was found that the addition of water had no effect on the equilibrium. This suggests that the DMSO and water molecules have similar roles in the prototropy of studied amides, i.e., the oxygen atoms in both molecules act as the base supporting deprotonation in the first step. Notably, a high reaction barrier was obtained for N-to-N (**A**-to-**E**) proton shift (Figure 3). The difference between these solvents is obvious, as water may deliver its own proton in the transition state, leading to the **E** tautomer but not the proton detached from amide, namely forming the *het*N⋯**H**—O(H)⋯**H**—N—(C=O) bridge (in equilibrium with *het*N**H**⋯O(H)—**H**⋯N—(C=O)). However, this detailed discussion is far beyond the scope of the current research.

### 2.2. Theoretical Calculations

#### 2.2.1. Tautomeric Equilibrium in Solvent

The theoretical calculations were performed with the ωB97X-D functional [17] and def2-TZVP basis set [18]. Such a choice of approach was based on the literature data for the tautomeric equilibrium in heterocyclic systems [17,19,20]. In the first step, the solvent effects were routinely included via the PCM implicit solvent model.

**Figure 4:**
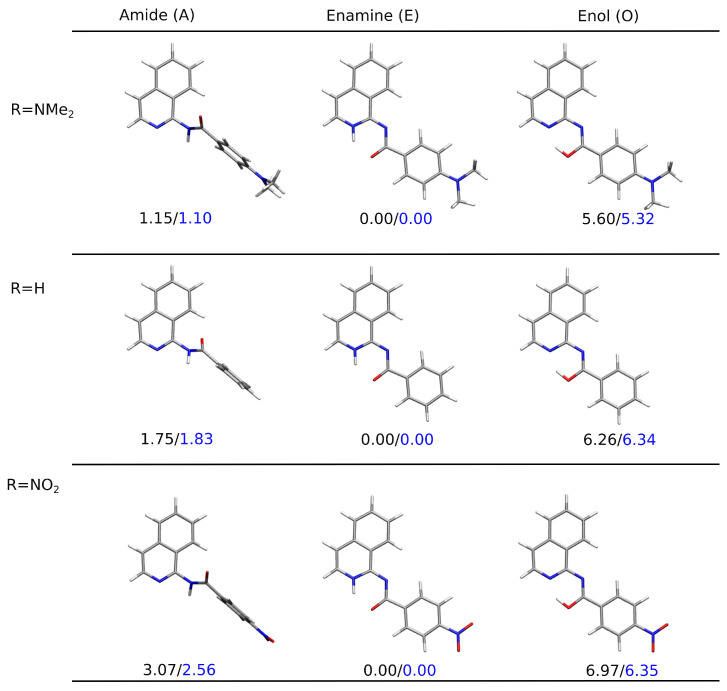
Optimized structures for the tautomeric forms of NMe${}_{2}$, H, and NO${}_{2}$-substituted derivatives (**1**, **5**, and **10**) together with the relative energies (in black) and the relative Gibbs free energies (in blue) in kcal/mol (ωB97X-D/def2-TZVP/PCM(DMSO) calculations).

Figure 4 presents the PCM-optimized lowest-energy structures for the tautomers of the three derivatives: the unsubstituted and the two bearing the strongest electron-donating and the strongest electron-accepting substituent in the analyzed sequence NMe${}_{2}$ and NO${}_{2}$, respectively. The clear preference for the planar enamine tautomer in the continuous solvent model was observed independent of the substituent present in the system when taking into account the relative Gibbs free energy and for plain electronic energy differences as well. The twisted amide tautomer in all these three molecules in PCM was characterized by their relative energy slightly higher than the so-called chemical accuracy. However, ΔG(A) equaled 3 kcal/mol at most for the nitro-derivative, indicating that the mixture of these two tautomers should be found in the sample at room temperature. The enol form, exhibiting the relative Gibbs free energy of the order of 5–7 kcal/mol, remained significantly less abundant in the experimental conditions (only trace amounts).

Yet, the implicit solvent model results did not easily follow the experimental findings based on signal integration in NMR spectra and their ratio presented in Table 1. The theoretical mixture composition determined from the Boltzmann distribution confirmed the increasing fraction of the enamine tautomer with the growing electron-accepting character of the substituent; nevertheless, the agreement was far from satisfactory when taking into account the prevailing form and relation of the two most abundant tautomers (Table 2). Therefore, improvements in the applied model are necessary in order to obtain a reasonable correspondence of theory to experiment. Two enhancements were tested: (*a*) the inclusion of the electron correlation effects for the tautomer and (*b*) the explicit solvent molecule involved in intermolecular hydrogen bonding with the respective tautomers. However, it needs to be underlined that the interaction of the oxygen atom in DMSO with the NH proton in **E** and **O** forms is believed to be weak due to the O/O lone pairs and O/heterocycle repulsion.

Because of the relatively small energy difference between the amide and enamine tautomers, the DFT functional choice was verified with a comparison of the corresponding relative Gibbs free energy data to those obtained with the approaches, including electron correlation effects (DLPNO-CCSD(T)/def2-TZVP, and CBS-QB3). The obtained relative Gibbs free energies are presented in Supplementary Information in Table S1. The hybrid complete basis set model in its CBS-QB3 variant [21,22] provided the same energetic order of tautomers and only slightly reduced energy differences between the forms. The DLPNO-CCSD(T) approach, on the other hand, maintained the reverse order of the lowest energy amide and enamine tautomers for all the investigated systems with respect to the DFT results, stabilizing the amide form over enamine by 1.57 to 2.57 kcal/mol already within the implicit solvent model. This order was reproduced even for the plain DLPNO-CCSD(T) energy differences with no zero-point vibrational and solvent corrections but with ΔE values lower than 3 kcal/mol in the most pronounced case of **10**. This indicates that the DLPNO-CCSD(T) approach can be a valuable alternative for the tautomeric equilibrium investigation; however, it needs to be further verified.

Routine calculations devoted to the tautomeric equilibrium are usually performed with continuum solvent models. However, the explicit inclusion of the solvent molecules forming hydrogen bonds with the solute, thus additionally stabilizing the electrostatic interaction or catalyzing the proton transfer process, proved vital in the case of tiny energetic differences, as observed in the present case. Such a microsolvation study is critical, particularly for solvents such as water, and is well recognized theoretically [19,20,23,24,25,26,27]. Nevertheless, systematic microsolvation studies for other solvents are scarce. Here, a single DMSO molecule was applied as an explicit solvent to demonstrate its essential role in tautomer stabilization (approach *b* mentioned above). We believe that its influence will be particularly substantial for systems creating relatively strong intermolecular hydrogen bonds, such as NH⋯O in the current study or OH⋯O. However, it is likely to be marginal for weak CH⋯O bonds, observed in 2-phenacylquinolines and 2-phenacylpyridines [1,9].

The composition of the reaction mixture for the investigated systems, presented in Table 2, together with the stability gathered in Table 3, strongly depended on the solvent model applied in the calculations. The direct comparison between the tautomers and derivatives explicitly interacting with the DMSO molecule was impeded by the different stable conformations and varying arrangement of the explicit solvent molecule(s) included in the calculations [20]. Considering the huge number of possible structures for the microsolvated clusters of these flexible molecules and the different solvent arrangements for the various derivatives, the observed tendencies for the relative tautomer energies resulted from the superposition of several factors, namely the isolated isoquinoline derivative structure, its interaction with the continuum solvent, and the pattern of the explicit DMSO molecules interacting with different parts of the isoquinoline either electrostatically, via hydrogen bond, or with the dispersion forces.

**Figure 5:**
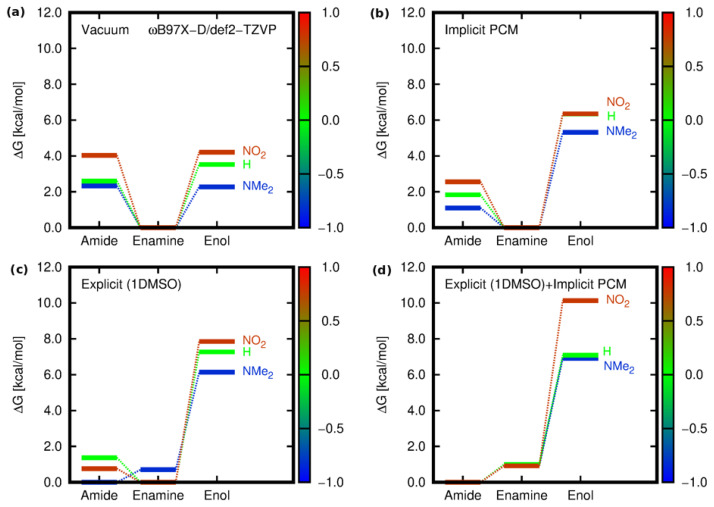
Relative Gibbs free energies for the tautomeric forms of **1**, **5**, and **10** [kcal/mol] estimated within the ωB97X-D/def2-TZVP approach for different environments: (**a**) isolated molecule in vacuum, (**b**) implicit solvent model (PCM) for DMSO, (**c**) explicitly included interaction of one solvent (DMSO) molecule with the analyzed derivative in vacuum, and (**d**) hybrid implicit (PCM)–explicit (DMSO) model (the color scale corresponds to the values of the Hammett constant of the substituent present in the phenyl ring).

The calculations performed in vacuum with isoquinoline derivative interacting with a single DMSO molecule decreased the Gibbs free energy difference between the amide and enamine tautomers; nevertheless, they still tend to point to the enamine (compare Figure S26). Yet, one can notice that the corresponding ΔG values (see Table 3 and Figure 5) were close to or even below the accuracy of the applied approach. Therefore, the unambiguous assignment of the most stable tautomer required special care. The possibility of the hydrogen bond formation between the solute and the solvent balanced the stability of the amide and enamine forms, and their corresponding energy difference became smaller than the chemical accuracy (about 1 kcal/mol). Additionally, the inspection of the structure for the analyzed solute:solvent complexes indicate that while in the case of **A** and **E**, the DMSO molecule tended to arrange close to the central part of the isoquinoline in order to benefit from the intermolecular hydrogen bond with the labile proton, in **O**, the intramolecular hydrogen bond stabilizing the derivative was too strong to allow conformational changes and open for the intermolecular solute:solvent H-bond. Thus, in the most stable enol rotamers, the planar molecular skeleton was preserved, and the solvent molecule flowed above the aromatic rings, interacting weakly and rather benefiting from dispersion interactions. The twisted enol skeleton, with the broken intramolecular H–bond and the N2–C1–N11–C12 dihedral angle close to 90${}^{\circ }$, allowed for the formation of the intermolecular hydrogen bond with the explicit solvent molecule; however, its energy seemed to be about 2 kcal/mol higher than for the planar enol arrangement (compare Figure S27).

**Figure 6:**
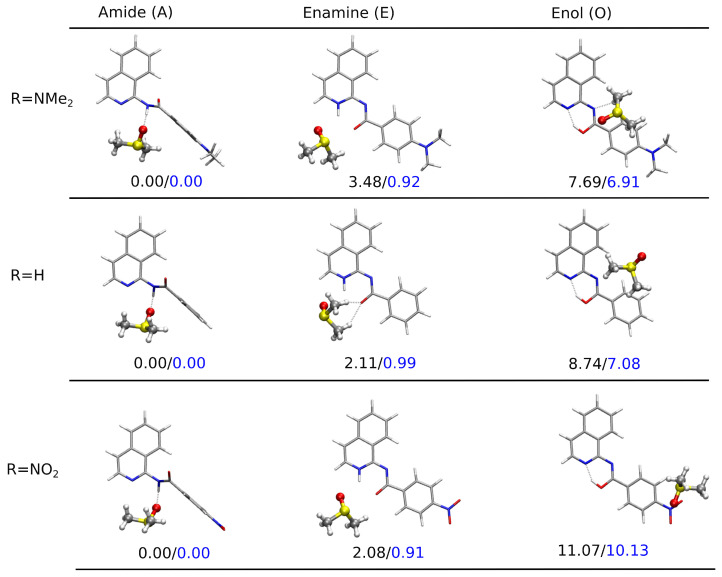
Optimized structures for the tautomeric forms of NMe${}_{2}$, H, and NO${}_{2}$-substituted derivatives (**1**, **5**,p and **10**) together with the relative Gibbs free energies in kcal/mol (ωB97X-D/def2-TZVP/PCM(DMSO) calculations for the complexes with the explicitly included one solvent molecule). The solute–solvent complex for the enamine tautomer of the unsubstituted R=H system **5** featured a different placement of DMSO molecule with respect to the N-H⋯O moiety than **1** and **10**. The configuration analogous to **1** and **10** was about 1.3 kcal/mol higher in energy. For the enol tautomers, the potential energy surface is significantly flatter with a huge number of shallow minima arising from a multitude of various positions of DMSO over or under the enol molecule plane in the case of all the derivatives; thus, a direct comparison of the different derivatives may not be straightforward, but qualitative conclusions can be made.

A similar scenario was observed in the hybrid implicit–explicit solvation model, and the corresponding optimized structures are presented in Figure 6. It can be easily observed that the position of the DMSO molecule was strongly dependent on the accessibility of the labile proton and significantly varied between the tautomers in this case as well. While for **A** and **E**, DMSO remained close to this proton and benefited from electrostatic interactions, in the case of **O**, the intramolecular hydrogen bond in the six-membered quasi-ring reduced the availability of the H13 proton for explicit solvent binding. Therefore, the relative energy of **O** grew to more than 6 kcal/mol, thus reducing its content in the mixture to traces only. The application of the hybrid implicit–explicit solvent model consistently indicated the best stability of the amide complex with DMSO in the case of all the substituents analyzed. However, the estimation of the mixture composition from the Boltzmann distribution provided a strong prevalence toward the amide tautomer (above 83% for all the substituents), while the experimental NMR considerations inverted the ratio from 3:1 in the case of the NMe${}_{2}$ derivative to 1:2 for the NO${}_{2}$-substituted one. Thus, it is evident that both implicit and explicit models significantly contribute to the tautomeric equilibrium analysis, but their adequacy for a detailed study may strongly depend on the system under consideration and the setup of the whole simulation approach. Nevertheless, the explicit solvent seemed to be mandatory to reproduce the experimental findings in the analyzed equilibrium.

The comparison of the three variants of the solvent models, namely implicit, explicit, and hybrid (a solute and one solvent molecule immersed in the continuum PCM solvent), together with the vacuum results for the three limiting cases, is presented in Figure 5. In vacuum and in the implicit PCM solvent, the most stable form was consequently and unequivocally the enamine (**E**). The similar relative energy of amide (**A**) and enol (**O**) in vacuum was disturbed in a continuous solvent, with a significant preference for the amide over the enol tautomer. Nevertheless, agreement with the experiment with respect to the most stable amide form could not be achieved until the inclusion of the explicit DMSO molecule interacting with the isoquinoline derivative.

#### 2.2.2. Barriers to Proton Transfer and Internal Rotation

In order to gain insight into the possibility of tautomer interconversion, the transition states for proton transfer and for the respective rotations along the C–N bonds were estimated using the ωB97X-D/def2-TZVP approach in the implicit solvent. Additionally, the exemplary structures of the 1:1 solute:explicit solvent complexes immersed in PCM were also considered. The explicit solvent calculations had to be carefully performed since the number of degrees of freedom was high, and particularly, the rotation involved a change in the position of the DMSO molecule. Therefore, the obtained energy barriers may consist not only of the desired contribution but also include some fraction of the energy difference arising from the solvent rearrangement. The corresponding energies and structures of **5** are presented in Figure 3. Since a detailed study of the explicitly represented DMSO molecule proved that its interaction with **E** and **O** forms was weak, the DMSO molecule could be excluded from the consideration of transition state calculations in the first approximation. This was supported by the fact that the total distance for the proton to change its bonding from NH to OH was very small and amounted to about 0.75 Å, while the distance between N2 and O13 atoms in the quasi-ring was equal to about 2.5 Å in the enol tautomer and 2.6 Å in the enamine tautomer (see Table S2).

Among the analyzed structures, a high energy barrier was observed for the proton transfer from the amide to enamine in its rotated conformation, exhibiting the relative Gibbs free energy for **5** of 6.97 kcal/mol in the implicit solvent. This barrier equal to 38.83 kcal/mol was prohibitively high, and proton transfer does not occur this way in practice. The high energy value suggested that the proton transfer in the polar DMSO solution may occur due to the solvent molecule in the following proposed path: (*a*) complex of **A** with DMSO, (*b*) a proton shift between NH and DMSO oxygen, (*c*) rotamerism within partially deprotonated isoquinoline (the intermolecular transition state), and (*d*) a proton shift between DMSO and the heterocyclic nitrogen atom with the simultaneous desolvation of the **E** tautomer. The proton transfer between the enamine and enol tautomers required a Gibbs free energy of 5.29 kcal/mol for **5**, thus indicating that this reaction was almost without barrier when proceeding from the enol to enamine. Such a tendency was retained for all the analyzed substituents (compare Figure 3). Furthermore, the rotation along the C-N bonds both in amides and enamines occurred with only a small energy cost, usually in the order of several kcal/mol. The explicit DMSO molecule included into the PCM considerations additionally stabilized the **A** and **E** tautomers with respect to the corresponding transition states; however, it did not change the relative energy scale.

#### 2.2.3. Non-Covalent Interactions

The atoms-in-molecule parameters allowed for the estimation of the strength of the hydrogen bond according to Espinosa [28]. For the intermolecular solute:solvent hydrogen bond in the case of the amide tautomer, the H11⋯O(DMSO) attraction grew in the order of increasing value of the substituent Hammett constant, from −7.21 kcal/mol for molecule **1** containing the dimethylamino group to −8.86 kcal/mol for system **10** with the nitro-substituent (compare Table S5). This tendency is fully in line with the experimental observations presented on the left panel of Figure 2 for the H11 chemical shift.

The enol tautomer exhibited a relatively strong intramolecular H-bond in both the implicit and explicit solvents, with its energy reaching almost −25 kcal/mol in PCM and usually stronger than −20 kcal/mol in the case of the explicit DMSO present. The N−H⋯O intramolecular interaction in the enamine form amounted to almost −15 kcal/mol for the implicit solvent and reduced to about −10 kcal/mol in the calculations including one explicit solvent molecule. Thus, the presence of explicit DMSO more strongly affected the intramolecular interactions in enamines than those in enols.

**Figure 7:**
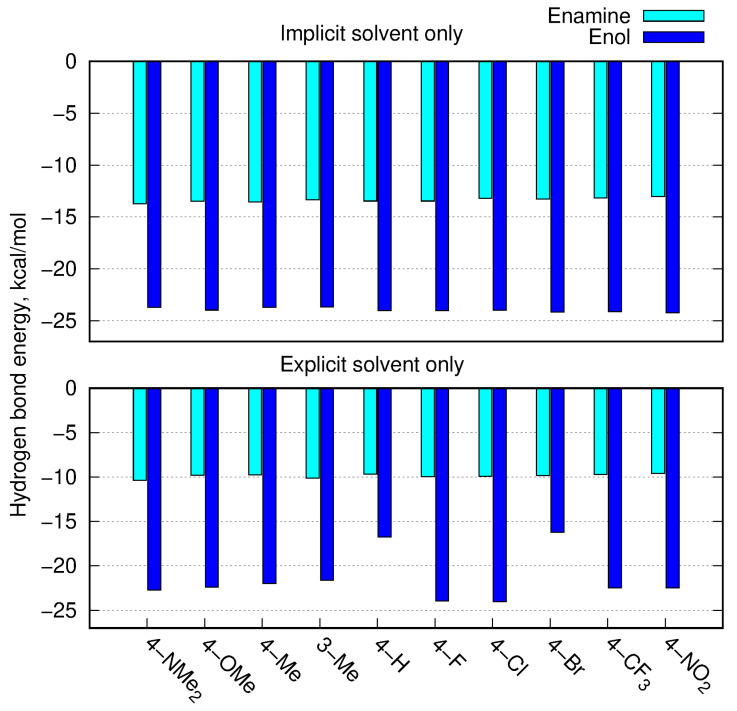
Estimation of intramolecular hydrogen bond energy (kcal/mol) in enamine and enol forms estimated according to Espinosa [28], for the ωB97X-D/def2-TZVP approach in the PCM solvent model (upper panel) and with the explicit solvent (lower panel).

Moreover, the explicit DMSO molecule remained in close contact with the enamine in the most stable complexes, and the strongest mutual attraction arose from two intermolecular interactions: O⋯H−N2 and O⋯H−C3. Both of these interactions were much weaker than the intramolecular H-bond and equaled about −3.5 and −2.5 kcal/mol, respectively. Additionally, the O20⋯HC in the critical point of the DMSO methyl groups’ (one or two symmetrically, depending on the system,) bond provided the stabilization of about 2.5 kcal/mol each (see Figure S29). The electron density values ρ and its laplacian are summarized in Supplementary Information (Table S6), while energy of intramolecular hydrogen bond is presented graphically in Figure 7.

In the explicit solvent calculations, the twisted enol tautomers could appear, where the intramolecular hydrogen bond was corrupted, and the stabilization resulted from the intermolecular solute:solvent hydrogen bond (see Figure S27). Such conformers were less stable than the planar ones benefiting from the intramolecular H-bonds, and the corresponding Gibbs free energy difference was about 3 kcal/mol. Taking into account the high relative Gibbs free energy of the enol tautomers with respect to the lowest amide tautomers in general, this twisted form is not expected to play an important role in the reaction mixture in standard conditions. However, since it can appear even in systematic studies supported with automatic tools such as CREST, and it strongly affects the NMR chemical shifts and H-bonding, its presence needs to be carefully examined.

SAPT0 energy for the explicit solute:solvent complexes significantly indicated a weaker interaction for the enol tautomer with DMSO than for the other two tautomers (Table S8). This **O** interaction was dominated by dispersion forces, particularly for the systems containing the ED substituents. The **A** and **E** complexes with DMSO were governed by electrostatic interactions. The total SAPT0 interaction was the strongest for amide (**A**) complexes and amounted to about 17–20 kcal/mol. These differences in the interaction energy of the tautomers with DMSO explain the influence of the explicit solvent model.

## 3. Materials and Methods

### 3.1. Synthesis

All substrates for the synthesis (amines, benzoyl chlorides, and benzoic acid esters) and solvents were obtained from commercial sources. The studied series of amides was obtained through a one-step synthesis using two methods (A and B) depending on substrate availability. The structure of the synthesized compounds was confirmed via ${}^{1}$H, ${}^{13}$C, and ${}^{19}$F (if reasonable) NMR. All the NMR spectra were recorded at 600 MHz using a Bruker spectrometer at 23 °C in DMSO-D${}_{6}$. The melting points were measured using a Stuart SMP50 digital melting-point instrument.

#### 3.1.1. Method A

To a solution of isoquinoline-1-amine (1 eq.) in dry THF (20 mL, under inert gas), NaH (2 eq.) was added and heated at 50 ${}^{\circ }$C for 45 min. After cooling, a respective benzoate ester (1 eq.) was added (in dry THF). The mixture was allowed to be heated under reflux overnight. The reaction was quenched (at 20 ${}^{\circ }$C) by adding an NH${}_{4}$Cl (4 eq.) solution (10 mL) in water. The solvent was evaporated, and the residue was extracted using a DCM/NaHCO${}_{3}$ water solution. After evaporation of the organic layer, the compound was purified through crystallization from ethanol.

#### 3.1.2. Method B

To a solution of isoquinoline-1-amine (1 eq.) and triethylamine (2 eq.) in dry THF (20 mL, −78 ${}^{\circ }$C, under inert gas), a respective benzoyl chloride (1 eq.) was added dropwise (in dry THF). The mixture was allowed to warm to room temperature and stirred overnight. The solvent was evaporated, and the residue was extracted using a DCM/NaHCO${}_{3}$ water solution. After evaporation of the organic layer, the compound was purified through recrystallization from ethanol.

**Compound 1, 4-NMe${}_{2}$** Method A. Yield 92%. mp 170–172 ${}^{\circ }$C, brown powder, ${}^{1}$H NMR (600 MHz, from TMS, DMSO-D${}_{6}$, forms **E** and **A** are labeled in NMR data): δ (ppm) 14.89 (**E**, s, 1H), 10.57 (**A**, s, 1H), 8.86 (**E**, d, 1H, *J* = 8.1 Hz), 8.35 (**A**, d, 1H, *J* = 6.2 Hz), 8.19 (**E**, d, 2H, *J* = 8.9 Hz), 7.98 (**A**, d, 1H, *J* = 7.7 Hz), 7.96 (**A**, d, 2H, *J* = 8.1 Hz), 7.91 (**A**, d, 1H, *J* = 8.9 Hz), 7.78 (**E**, m 2H), 7.75 (**A**, d, 1H, *J* = 5.0 Hz), 7.76 (**A**, m, 1H), 7.67 (**E**, d, m, 2H), 7.61 (**A**, m, 1H), 7.09 (**E**, d, 1H, *J* = 6.9 Hz), 7.77 (**A**, d, 2H, *J* = 9.2 Hz), 7.74 (**E**, d, 2H, *J* = 9.2 Hz), 3.01 (**A**, s, 6H), 3.00 (**E**, s, 6H). ${}^{13}$C NMR (150 MHz, from TMS, DMSO-D${}_{6}$): δ (ppm) 177.4, 167.1, 156.9, 153.2, 153.0, 151.9, 141.5, 137.7, 137.4, 133.4, 131.4, 131.2, 13.9, 130.1, 128.4, 128.0, 127.6, 127.1, 127.0, 126.7, 126.0, 125.9, 120.5, 120.1, 111.3, 111.2, 111.2, 111.0.

**Compound 2, 4-OMe** Method A. Yield 57%. mp 134–136 ${}^{\circ }$C, white powder, ${}^{1}$H NMR (600 MHz, from TMS, DMSO-D${}_{6}$): δ (ppm) 14.86 (**E**, s, 1H), 10.83 (**A**, s, 1H), 8.90 (**E**, d, 1H, *J* = 7.9 Hz), 8.37 (**A**, d, 1H, *J* = 5.0 Hz), 8.31 (**E**, d, 2H, *J* = 7.9 Hz), 8.06 (**A**, d, 1H, *J* = 8.9 Hz), 8.00 (**A**, d, 2H, *J* = 7.8 Hz), 7.94 (**A**, d, 1H, *J* = 8.7 Hz), 7.84 (**E**, m 2H), 7.78 (**A**, d, 1H, *J* = 5.0 Hz), 7.76 (**A**, m, 1H), 7.68 (**E**, d, m, 2H), 7.63 (**A**, m, 1H), 7.18 (**E**, d, 1H, *J* = 6.8 Hz), 7.08 (**A**, d, 2H, *J* = 8.8 Hz), 7.02 (**E**, d, 2H, *J* = 8.6 Hz), 3.85 (**A**, s, 3H), 3.83 (**E**, s, 3H). ${}^{13}$C NMR (150 MHz, from TMS, DMSO-D${}_{6}$): δ (ppm) 176.7, 166.8, 162.8, 162.5, 157.1, 151.4, 141.5, 133.7, 131.4, 131.0, 130.5, 128.4, 128.2, 127.8, 127.2, 127.1, 126.5, 120.4, 114.2, 113.8, 111.7, 55.9, 55.8.

**Compound 3, 4-Me** Method A. Yield 78%. mp 255–258 (dec.) ${}^{\circ }$C, brown crystals, ${}^{1}$H NMR (600 MHz, from TMS, DMSO-D${}_{6}$): δ (ppm) 14.86 (**E**, s, 1H), 10.88 (**A**, s, 1H), 8.91 (**E**, d, 1H, *J* = 8.4 Hz), 8.37 (**A**, d, 1H, *J* = 5.5 Hz), 8.25 (**E**, d, 2H, *J* = 8.1 Hz), 8.01 (**A**, d, 1H, *J* = 8.4 Hz), 7.98 (**A**, d, 2H, *J* = 8.4 Hz), 7.95 (**A**, d, 1H, *J* = 8.6 Hz), 7.85 (**E**, m 2H), 7.79 (**A**, d, 1H, *J* = 5.5 Hz), 7.78 (**A**, m, 1H), 7.72 (**E**, d, m, 2H), 7.63 (**A**, m, 1H), 7.36 (**A**, d, 2H, *J* = 8.4 Hz), 7.33 (**E**, d, 2H, *J* = 7.8 Hz), 7.21 (**E**, d, 1H, *J* = 6.9 Hz), 2.40 (**A**, s, 3H), 2.38 (**E**, s, 3H). ${}^{13}$C NMR (150 MHz, from TMS, DMSO-D${}_{6}$): δ (ppm) 176.9, 167.2, 157.3, 151.3, 142.5, 141.8, 141.6, 137.7, 137.5, 136.2, 133.7, 131.5, 131.0, 129.6, 129.5, 129.2, 128.6, 128.4, 128.2, 127.9, 127.2, 127.2, 127.1, 126.4, 125.7, 124.8, 120.5, 112.0, 21.5.

**Compound 4, 3-Me** Method A. Yield 71%. mp 139–141 ${}^{\circ }$C, brown powder, ${}^{1}$H NMR (600 MHz, from TMS, DMSO-D${}_{6}$): δ (ppm) 14.86 (**E**, s, 1H), 10.88 (**A**, s, 1H), 8.93 (**E**, d, 1H, *J* = 8.5 Hz), 8.38 (**A**, d, 1H, *J* = 5.6 Hz), 8.17 (**E**, m, 2H), 8.01 (**A**, d, 1H, *J* = 7.4 Hz), 7.97 (**A**, d, 1H, *J* = 8.4 Hz), 7.90 (**A**, m, 1H), 7.88 (**A**, m, 1H), 7.86 (**E**, m, 2H), 7.80 (**A**, d, 1H, *J* = 5.6 Hz), 7.78 (**A**, m, 1H), 7.72 (**E**, m, 2H), 7.64 (**A**, m, 1H), 7.44 (**A**, m, 2H), 7.37 (**E**, m, 2H), 7.23 (**E**, d, 1H, *J* = 7.0 Hz), 2.41 (**E**, s, 3H), 2.40 (**A**, s, 3H). ${}^{13}$C NMR (150 MHz, from TMS, DMSO-D${}_{6}$): δ (ppm) 177.0, 167.4, 157.3, 151.2, 141.6, 138.8, 138.3, 137.7, 134.3, 133.8, 133.0, 132.5, 131.0, 129.9, 128.8, 128.4, 128.3, 127.9, 127.2, 126.8, 126.4, 125.6, 124.7, 120.5, 112.2, 21.6, 21.4.

**Compound 5, H** Method A. Yield 65%. mp 93–95 ${}^{\circ }$C, brown powder, ${}^{1}$H NMR (600 MHz, from TMS, DMSO-D${}_{6}$): δ (ppm) 14.85 (**E**, s, 1H), 10.88 (**A**, s, 1H), 8.93 (**E**, d, 1H, *J* = 8.2 Hz), 8.39 (**A**, d, 1H, *J* = 6.0 Hz), 8.36 (**E**, d, 2H, *J* = 7.7 Hz), 8.08 (**A**, d, 2H, *J* = 6.9 Hz), 8.01 (**A**, d, 1H, *J* = 8.2 Hz), 7.98 (**A**, d, 1H, *J* = 8.6 Hz), 7.87 (**E**, m, 2H), 7.80 (**A**, d, 1H, *J* = 6.0 Hz), 7.78 (**A**, m, 1H), 7.73 (**E**, m, 1H), 7.64 (m), 7.55 (**A**, m, 2H), 7.52 (**E**, m, 2H), 7.23 (**E**, d, 1H *J* = 6.9 Hz). ${}^{13}$C NMR (150 MHz, from TMS, DMSO-D${}_{6}$): δ (ppm) 176.8, 167.3, 157.3, 151.1, 141.6, 138.8, 137.7, 137.5, 134.3, 133.8, 132.5, 131.9, 131.0, 129.4, 129.0, 128.6, 128.5, 128.3, 127.9, 127.3, 127.2, 126.4, 125.7, 124.7, 120.6, 112.3.

**Compound 6, 4-F** Method A. Yield 70%. mp 151–153 ${}^{\circ }$C, brown powder, ${}^{1}$H NMR (600 MHz, from TMS, DMSO-D${}_{6}$): δ(ppm) 14.83 (**E**, s, 1H), 11.02 (**A**, s, 1H), 8.92 (**E**, d, 1H, *J* = 8.9 Hz), 8.41 (**E**, m, 2H), 8.38 (**A**, d, 1H, *J* = 5.9 Hz), 8.14 (**A**, m, 2H), 8.01 (**A**, d, 1H, *J* = 8.2 Hz), 7.97 (**A**, d, 1H, *J* = 8.5 Hz), 7.87 (**E**, m 2H), 7.80 (**A**, d, 1H, *J* = 5.9 Hz), 7.79 (**A**, m, 1H), 7.72 (**E**, m, 2H), 7.64 (**A**, m, 1H), 7.39 (**A**, t, 2H, *J* = 8.8 Hz), 7.30 (**E**, t, 2H, *J* = 8.8 Hz), 7.24 (**E**, d, 1H, *J* = 6.8 Hz). ${}^{19}$F NMR (376 MHz, from CFCl${}_{3}$, DMSO-D${}_{6}$): δ (ppm) −109.1, −110.4. ${}^{13}$C NMR (150 MHz, from TMS, DMSO-D${}_{6}$): δ (ppm) 175.6, 166.3, 165.7, 165.6, 164.0, 164.0, 157.3, 151.0, 141.6, 137.7, 137.6, 135.4, 133.8, 132.1, 132.0, 131.3, 131.2, 131.1, 128.4, 128.3, 127.9, 127.3, 127.2, 127.1, 126.4, 125.6, 124.7, 120.6, 116.0, 115.9, 115.5, 115.3, 112.3.

**Compound 7, 4-Cl** Method A. Yield 79%. mp 177–179 ${}^{\circ }$C, powder ${}^{1}$H NMR (600 MHz, from TMS, DMSO-D${}_{6}$): δ (ppm) 14.80 (**E**, s, 1H), 11.03 (**A**, s, 1H), 8.91 (**E**, d, 1H, *J* = 8.1 Hz), 8.38 (**A**, d, 1H, *J* = 5.4 Hz), 8.36 (**E**, d, 2H, *J* = 8.1 Hz), 8.09 (**A**, d, 2H, *J* = 8.5 Hz), 8.01 (**A**, d, 1H, *J* = 8.2 Hz), 7.98 (**A**, d, 1H, *J* = 8.8 Hz), 7.89 (**E**, m 2H), 7.80 (**A**, d, 1H, *J* = 5.4 Hz), 7.78 (**A**, m, 1H), 7.76 (**E**, d, m, 2H), 7.71 (**A**, m, 1H), 7.63 (**A**, d, 2H, *J* = 8.5 Hz), 7.55 (**E**, d, 2H, *J* = 8.5 Hz), 7.25 (**E**, d, 1H, *J* = 6.9 Hz). ${}^{13}$C NMR (150 MHz, from TMS, DMSO-D${}_{6}$): δ (ppm) 175.6, 166.3, 157.3, 150.9, 141.6, 137.7, 137.7, 137.6, 137.4, 136.8, 133.9, 133.1, 131.3, 131.1, 130.4, 129.1, 128.6, 128.4, 128.3, 128.0, 127.3, 127.2, 127.1, 126.3, 125.6, 124.6, 120.6, 112.54.

**Compound 8, 4-Br** Method A. Yield 75%. mp 209–211 ${}^{\circ }$C, white powder, ${}^{1}$H NMR (600 MHz, from TMS, DMSO-D${}_{6}$): δ (ppm) 14.80 (**E**, s, 1H), 11.03 (**A**, s, 1H), 8.91 (**E**, d, 1H, *J* = 8.3 Hz), 8.38 (**A**, d, 1H, *J* = 5.5 Hz), 8.28 (**E**, d, 2H, *J* = 8.3 Hz), 8.01 (**A**, d, 2H, *J* = 8.3 Hz), 7.97 (**A**, d, 1H, *J* = 8.3 Hz), 7.87 (**E**, m 2H), 7.80 (**A**, d, 1H, *J* = 5.5 Hz), 7.77 (**A**, m, 1H), 7.71 (**E**, d, m, 2H), 7.64 (**A**, m, 1H), 7.77 (**A**, d, 2H, *J* = 8.3 Hz), 7.69 (**E**, d, 2H, *J* = 8.3 Hz), 7.26 (**E**, d, 1H, *J* = 6.9 Hz). ${}^{13}$C NMR (150 MHz, from TMS, DMSO-D${}_{6}$): δ (ppm) 175.7, 166.5, 157.3, 150.9, 141.6, 138.0, 137.7, 137.6, 133.9, 133.5, 132.0, 131.6, 131.5, 131.1, 130.6, 128.5, 128.3, 128.0, 127.3, 127.2, 127.2, 126.3, 125.8, 128.6, 124.6, 120.6, 112.6.

**Compound 9, 4-CF${}_{3}$** Method A. Yield 71%. mp 177–180 ${}^{\circ }$C, white crystals, ${}^{1}$H NMR (600 MHz, from TMS, DMSO-D${}_{6}$): δ (ppm) 14.85 (**E**, s, 1H), 11.26 (**A**, s, 1H), 8.96 (**E**, s, 1H), 8.55, 8.40, 8.31, 8.03, 7.90, 7.72, 7.32. ${}^{19}$F NMR (376 MHz, from CFCl${}_{3}$, DMSO-D${}_{6}$): δ (ppm) −62.1, −62.3. ${}^{13}$C NMR (150 MHz, from TMS, DMSO-D${}_{6}$): δ (ppm) 175.1, 166.3, 157.3, 150.6, 142.6, 141.6, 138.1, 137.7, 134.0, 131.2, 130.0, 129.5, 128.3, 127.2, 126.3, 125.7, 123.7, 120.7, 113.0, 56.5.

**Compound 10, 4-NO${}_{2}$** Method B. Yield 55%. mp 238–240 ${}^{\circ }$C, yellow powder ${}^{1}$H NMR (600 MHz, from TMS, DMSO-D${}_{6}$): δ (ppm) 14.8 (**E**, s, 1H), 11.29 (**A**, s, 1H), 8.95 (**E**, d, 1H, *J* = 8.3 Hz), 8.57 (**E**, d, 1H, *J* = 8.3 Hz), 8.39 (**A**, d, 2H, *J* = 5.8 Hz), 8.33 (**E**, d, 1H, *J* = 8.3 Hz), 8.27 (**E**, d, 2H, *J* = 8.3 Hz), 8.03 (**A**, d, 1H, *J* = 6.6 Hz), 7.91 (**E**, m 2H), 7.82 (**E**, m, 2H), 7.75 (**A**, m, 2H), 7.66 (**A**, m, 1H), 7.34 (**E**, d, 1H, *J* = 6.6 Hz). ${}^{13}$C NMR (150 MHz, from TMS, DMSO-D${}_{6}$): δ (ppm) 174.4, 165.9, 157.3, 150.5, 149.9, 149.7, 144.5, 141.6, 140.1, 137.7, 134.1, 131.2, 130.6, 130.0, 128.5, 128.1, 127.3, 127.3, 126.2, 125.5, 124.4, 124.1, 123.8, 120.8, 113.3.

### 3.2. Technical Details for Theoretical Calculations

The initial forms of **1–10** derivative tautomers were selected as the lowest energy configurations generated from the meta-dynamic simulation supported with semiempirical tight-binding calculations by the procedure available in the conformer–rotamer ensemble sampling tool (CREST), as implemented in the xTB program [29,30,31]. Furthermore, the obtained structures were reoptimized with the ωB97X-D/def2-TZVP method. Def2-reoptimized Ahlrichs basis sets were chosen for the present study due to their design for DFT calculations [18], while the long-range corrected ωB97X-D functional of Chai and Head–Gordon [32] containing the empirical dispersion correction was applied for its superior performance for non-bonded systems and good overall description of thermochemical quantities [17].

The solvent effects were routinely included in the continuum solvent model, using its IEF-PCM variant. Due to the vital presence of the solvent in the experimentally analyzed samples and earlier proofs of the crucial impact of microsolvation on the theoretical calculations of the tautomeric equilibria in organic systems [20], the solvent effects were also modeled with the inclusion of the single explicit dimethylsulfoxide molecule. Then, the initial position of DMSO was selected to enable intermolecular hydrogen bond formation with the solute. Because of the huge number of degrees of freedom in such a complex, the most important lowest-energy configurations were determined again with the meta-dynamics run performed within the CREST program [29,30,31]. This procedure provides hundreds of possible solute:solvent complexes with low energy, that may be important components of the reaction mixture in standard conditions. Next, the lowest energy complexes (up to 300 for the enol tautomers of **1**, **5**, and **10**) were reoptimized using the ωB97X-D/def2-TZVP approach, and after their careful examination, only the lowest energy one was selected for further analysis.

Additionally, since the single solvent molecule may not fully reproduce the bulk solvent influence, a hybrid explicit–implicit approach was applied, including the single explicit DMSO molecule in the PCM cavity, together with the isoquinoline derivative. The character of the stationary points on the potential energy surface was verified through the harmonic vibrational analysis. The influence of the electron correlation effects was confirmed using DLPNO-CCSD(T)/def2-TZVPP calculations. Because of the lack of available forces and solvent effects for this approach, the corresponding corrections to the energy of the molecule in a vacuum were added, as estimated at the DFT level. The thermochemistry of the equilibrium was also validated with the hybrid CBS-QB3 approach, developed with particular care to the thermodynamic properties description [21,22]. The CBS-QB3 scheme benefitted from the B3LYP-optimized geometries and frequencies, and MP4 and QCISD energy corrections extrapolated to the complete basis set limit.

The energy barriers for the proton transfer and intramolecular rotation were estimated using the ωB97X-D/def2-TZVP approach in PCM and for the exemplary complexes in the explicit solvent model as well. The transition state search was carried out as a Berny optimization process starting from the most probable transition state structure and confirmed with Hessian calculations and a thorough inspection of the imaginary frequency obtained. For the rotation along the single bonds, the initial transition state structures were taken from a relaxed scan performed using the ωB97X-D/def2-TZVP approach.

The NMR chemical shifts were obtained from GIAO magnetic shielding tensors, and the AIM properties were calculated at the same level of theory. The interaction energy and its components for the explicit solute:solvent complexes were determined using the SAPT0/def2-TZVPD approach, implemented in the Psi4 package [33,34,35,36]. All optimizations, chemical shift calculations, and CBS-QB3 calculations were performed with the Gaussian16 package of programs [37], and DLPNO-CCSD(T) calculations were carried out in Orca 4.0 [38]. An atom-in-molecule study was carried out with AIMAll [39].

## 4. Conclusions

The present study provided a detailed analysis of the tautomeric equilibrium in the series of 1-benzamidoisoquinoline derivatives. A controlled shift in the tautomeric equilibrium in the mixture could be observed due to the substituent effect. The computational study clearly showed that the proper reproduction of the relative molar fractions of different isomers for this type of system required the inclusion of the explicit solvent. The conventional, routinely applied implicit solvent models artificially overestimated the stability of the enamine tautomer, clearly suffering from the lack of an intermolecular solute:solvent hydrogen bond. Nevertheless, the explicit solvent molecule introduced additional degrees of freedom to the investigated complexes, and thus the total stability of the isoquinoline in DMSO was affected not only by the stability of the molecule itself but also, to a high extent, by the optimal arrangement of the solvent. Therefore, it needs to be underlined that for systems of this type, prone to strong H-bond formation, the *ab initio* study of the tautomeric equilibrium is far from routine and requires particular care in order to avoid qualitative errors.

## Figures and Tables

**Table 1:** ${}^{1}$H NMR chemical shifts (δ, ppm) for the selected protons in tautomers of compounds **1**–**10** in DMSO-D${}_{6}$ and their content (last column).

Comp. (R)	$\sigma {\;}^{a}$	δ(H2)	δ(H11)	δ(H3)	δ(H9)	Fraction (%)
**1E** (4-NMe${}_{2}$)	−0.83	14.87		8.86	7.09	26
**1A**			10.53	8.35	7.91	74
**2E** (4-OMe)	−0.27	14.86		8.90	7.18	35
**2A**			10.77	8.37	7.94	65
**3E** (4-Me)	−0.17	14.89		8.93	7.21	33
**3A**			10.84	8.37	7.95	67
**4E** (3-Me)	−0.07	14.98		8.93	7.22	32
**4A**			10.88	8.38	7.97	68
**5E** (4-H)	0.00	14.83		8.92	7.23	35
**5A**			10.96	8.39	7.98	65
**6E** (4-F)	0.06	14.80		8.92	7.24	42
**6A**			10.98	8.38	7.97	58
**7E** (4-Cl)	0.23	14.83		8.91	7.25	48
**7A**			11.03	8.38	7.98	52
**8E** (4-Br)	0.23	14.83		8.91	7.26	48
**8A**			11.03	8.38	7.97	52
**9E** (4-CF${}_{3}$)	0.54	14.85		8.94	7.32	54
**9A**			11.20	8.40	8.03	46
**10E** (4-NO${}_{2}$)	0.78	14.82		8.95	7.34	62
**10A**			11.29	8.39	8.03	38

^*a*^ Hammett substituent constant.

**Table 2:** The percentage fraction of the different tautomers of **1** and **10** in the mixture from the theoretical DFT calculations (boldfaced is the best correspondence between the experiment presented in Table 1 and the theoretical estimation).

	ωB97X-D/def2-TZVP											
	Vacuum			Implicit			Explicit			Hybrid		
Substituent	A	E	O	A	E	O	A	E	O	A	E	O
**1** (4-NMe${}_{2}$)	2	96	2	13	87	0	**77**	**23**	0	83	17	0
**10** (4-NO${}_{2}$)	0	100	0	1	99	9	**21**	**79**	0	83	17	0

**Table 3:** Relative Gibbs free energy (kcal/mol) with respect to the lowest energy amide (**A**) tautomer for the implicit solvent model PCM, explicit solvent model, and the hybrid implicit–explicit solvent model with single explicit DMSO molecule.

	Implicit Solvent			Explicit Solvent			Explicit–Implicit Solvent		
Compound	A	E	O	A	E	O	A	E	O
**1** (4-NMe${}_{2}$)	1.10	0.00	5.32	0.00	0.70	6.14	0.00	0.92	6.91
**2** (4-OMe)	2.56	0.00	5.59	1.45	0.00	7.99	0.00	1.72	8.43
**3** (4-Me)	2.85	0.00	6.78	0.95	0.00	8.15	0.00	1.69	7.71
**4** (3-Me)	1.42	0.00	5.43	0.14	0.00	6.75	0.00	2.33	6.36
**5** (H)	1.83	0.00	6.34	1.37	0.00	7.27	0.00	0.99	7.08
**6** (4-F)	2.78	0.00	6.17	1.09	0.00	7.58	0.00	0.46	6.82
**7** (4-Cl)	2.68	0.00	5.88	0.65	0.00	7.74	0.00	0.64	6.97
**8** (4-Br)	2.67	0.00	5.97	0.41	0.00	8.30	0.00	1.13	7.30
**9** (4-CF${}_{3}$)	0.35	0.00	4.34	0.60	0.00	8.19	0.00	2.54	8.41
**10** (4-NO${}_{2}$)	2.56	0.00	6.35	0.76	0.00	7.85	0.00	0.91	10.13

## Data Availability

The data presented in this study are available in supplementary material. The geometry of molecules obtained during calculations are available upon request form authors.

## Supplementary Materials

The following supporting information can be downloaded at: https://www.mdpi.com/article/10.3390/molecules28031101/s1, Supplementary Information contains the raw NMR spectra, the additional information on theoretical calculation results. References [28,34,35,36,40] are cited in the supplementary materials.
