# Supplementary material for: Tautomeric Equilibrium in 1-Benzamidoisoquinoline Derivatives
Source: Molecules. 2023 Jan 22;28(3):1101. doi: 10.3390/molecules28031101 (PMC9920963; doi:10.3390/molecules28031101)
Supplement: Supplementary file 1 [file molecules-28-01101-s001.zip › molecules-2165519-supplementary.pdf]

Supporting Information: Tautomeric equilibrium of 1-benzamidopyridines

Patryk Rybczyński,*,† Anna Kaczmarek-Kędziera,† Alex Iglesias-Reguant,‡ Damian Plażuk,¶ and Borys Ośmiałowski*,†

†*Faculty of Chemistry, Nicolaus Copernicus University, Gagarina 7, PL-87100 Toruń, Poland*

‡*Faculty of Chemistry, Nicolaus Copernicus University, Gagarina 7, PL-87100 Toruń, Poland; Institute of Computational Chemistry and Catalysis and Department of Chemistry, University of Girona, Campus de Montilivi, 17003 Girona, Catalonia, Spain*

¶*Laboratory of Molecular Spectroscopy, Department of Organic Chemistry, Faculty of Chemistry, University of Łódź, ul. Tamka 12, 91-403 Łódź, Poland*

E-mail: pat_ryb@doktorant.umk.pl; borys.osmialowski@umk.pl

List of Figures

S1 The ^{1}H NMR spectrum of compound **1** S-5
S2 The ^{13}C NMR spectrum of compound **1** S-6
S3 The ^{1}H NMR spectrum of compound **2** S-7
S4 The ^{13}C NMR spectrum of compound **2** S-8
S5 The ^{1}H NMR spectrum of compound **3** S-9
S6 The ^{13}C NMR spectrum of compound **3** S-10
S7 The ^{1}H NMR spectrum of compound **4** S-11
S8 The ^{13}C NMR spectrum of compound **4** S-12
S9 The ^{1}H NMR spectrum of compound **5** S-13
S10 The ^{13}C NMR spectrum of compound **5** S-14
S11 The ^{1}H NMR spectrum of compound **6** S-15
S12 The ^{13}C NMR spectrum of compound **6** S-16
S13 The ^{19}F NMR spectrum of compound **6** S-17
S14 The ^{1}H NMR spectrum of compound **7** S-18
S15 The ^{13}C NMR spectrum of compound **7** S-19
S16 The ^{1}H NMR spectrum of compound **8** S-20
S17 The ^{13}C NMR spectrum of compound **8** S-21
S18 The ^{1}H NMR spectrum of compound **9** S-22
S19 The ^{13}C NMR spectrum of compound **9** S-23
S20 The ^{19}F NMR spectrum of compound **9** S-24
S21 The ^{1}H NMR spectrum of compound **9** S-25
S22 The ^{13}C NMR spectrum of compound **9** S-26
S23 The reoptimized lowest energy structures of the **1** with implicit PCM solvent model (the relative Gibbs free energies in kcal/mol; the lowest energy tautomer in the blue circle) S-28

S24 The reoptimized lowest energy structures of the **5** with implicit PCM solvent model (the relative Gibbs free energies in kcal/mol; the lowest energy tautomer in the blue circle) S-29
S25 The reoptimized lowest energy structures of the **10** with implicit PCM solvent model (the relative Gibbs free energies in kcal/mol; the lowest energy tautomer in the blue circle) S-30
S26 The optimized solute:solvent complexes in vacuum together with the relative energy ΔE (in black) and the relative Gibbs free energy ΔG (in blue) [kcal/mol] S-31
S27 Optimized **5** enol tautomer with the twisted structure interacting with the explicit DMSO molecule S-32
S28 Molecular graphs explicit interaction of (a) **1**, (b) **5** and (c) **10** amide tautomer with DMSO molecule S-34
S29 Molecular graphs explicit interaction of (a) **1**, (b) **5** and (c) **10** enamine tautomer with DMSO molecule S-35
S30 Molecular graphs explicit interaction of (a) **1**, (b) **5** and (c) **10** enole tautomer with DMSO molecule S-35
S31 Correlation between Hammett substituent constant and ^{1}H NMR shifts of protons H9 (form **E** - a and **A** - b) and H3 (form **E** - c and **A** - d) S-36

List of Tables

S1 Relative Gibbs free energies for **1**, **5** and **10** tautomers in various approaches in PCM [kcal/mol] (the DLPNO-CCSD(T) corrected for solvent effects and zero-point energy with the DFT contributions) S-27
S2 Distances in the quasi-ring formed by the intramolecular hydrogen bonds for **1**, **5** and **10** tautomers in ωB97X-D/def2TZVP/PCM [Å] S-32

S3 Chemical shifts δ in ppm for ^{1}H NMR ωB97X-D/def2-TZVP S-33
S4 AIM parameters: electron density ρ, its laplacian $\bigtriangledown^2\rho$, total energy density H_b[S1] in a. u. for the indicated bond critical points and hydrogen-bond energy $E(HB)$ in kcal/mol, according to Espinosa,[S2] estimated in the implicit solvent model within the ωB97X-D/def2-TZVP approach for enamine and enol tautomers S-34
S5 AIM parameters: electron density ρ, its laplacian $\bigtriangledown^2\rho$ and total energy density H_b[S1] in a. u. for selected bond critical points (BCP) and hydrogen-bond energy $E(HB)$ in kcal/mol, according to Espinosa,[S2] estimated in the explicit solvent model within the ωB97X-D/def2-TZVP approach for amide tautomer of **1**, **5** and **10** (H(DMSO:1) and H(DMSO:2) denote the hydrogens from the DMSO methyl groups; compare the molecular graphs in Fig. S28) S-37
S6 AIM parameters: electron density ρ, its laplacian $\bigtriangledown^2\rho$ and total energy density H_b[S1] in a. u. for selected bond critical points (BCP) and hydrogen-bond energy $E(HB)$ in kcal/mol, according to Espinosa,[S2] estimated in the explicit solvent model within the ωB97X-D/def2-TZVP approach for enamine and enamine tautomers of **1**, **5** and **10** (H(DMSO:1) and H(DMSO:2) denote the hydrogens from the DMSO methyl groups; compare the molecular graphs in Figs. S29 and S30) S-38
S7 SAPT0/def2-TZVPD interaction energy and its components [kcal/mol] for the analyzed systems (last column, D/E, indicates the dispersion-to-electrostatic ratio); detailed description of the SAPT0 components can be found in Refs.[S3–S5] S-39

NMR and mass spectra

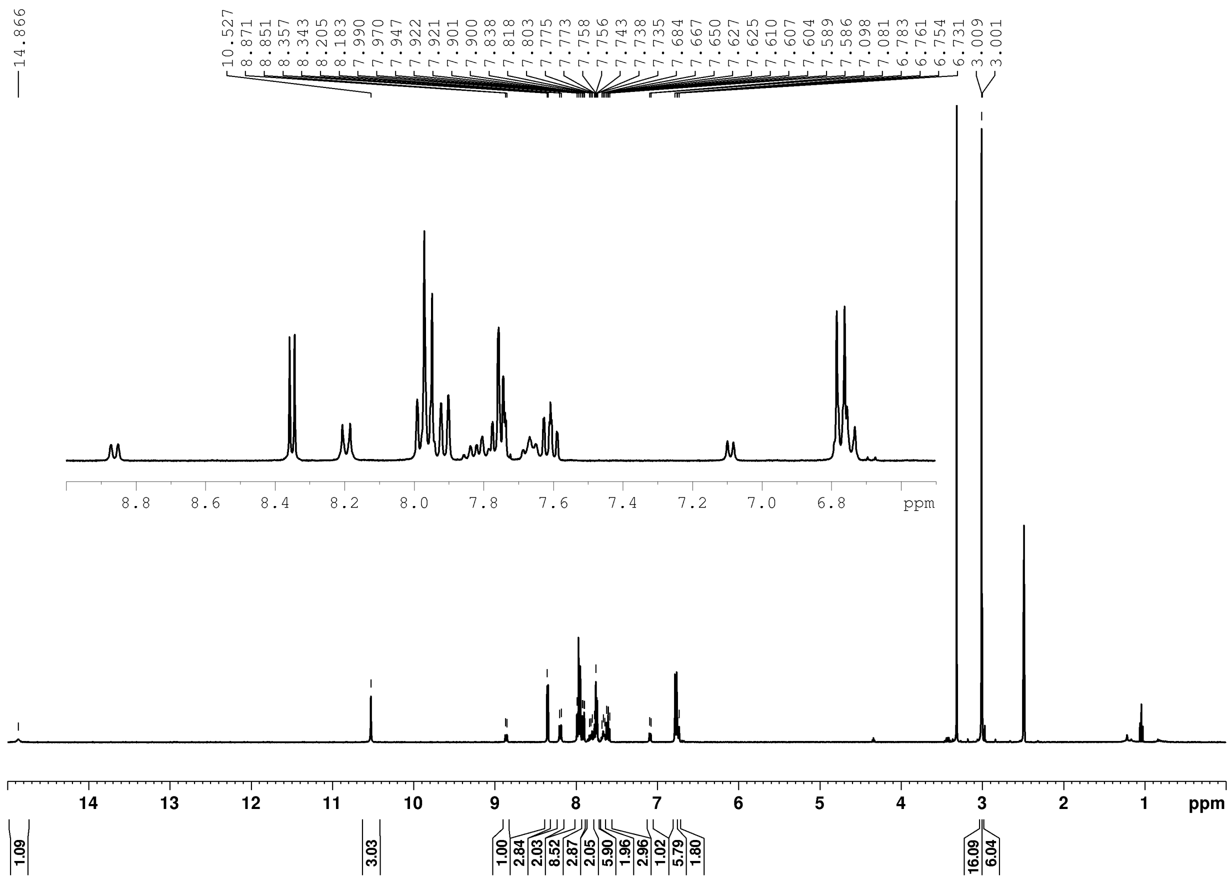

Figure S1: The ^{1}H NMR spectrum of compound **1**

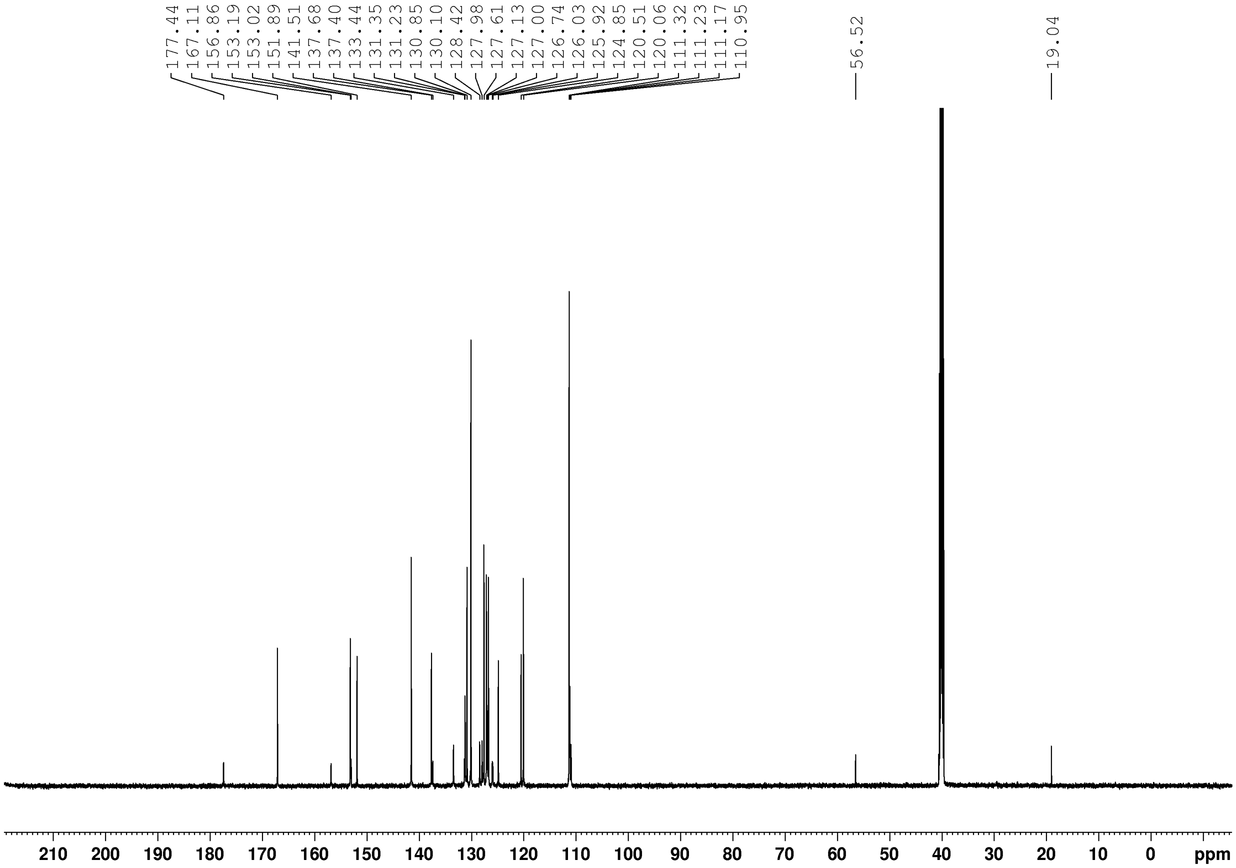

Figure S2: The ^{13}C NMR spectrum of compound **1**

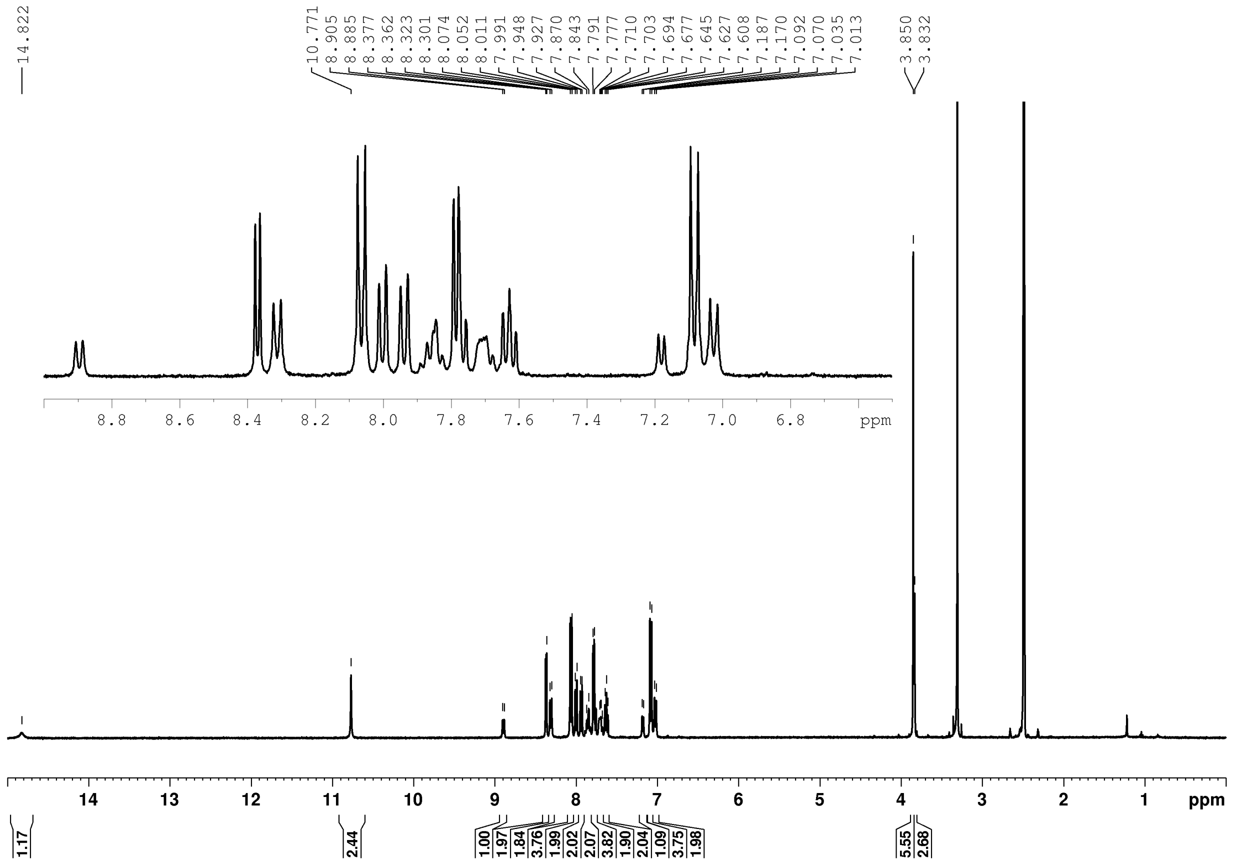

Figure S3: The ^{1}H NMR spectrum of compound **2**

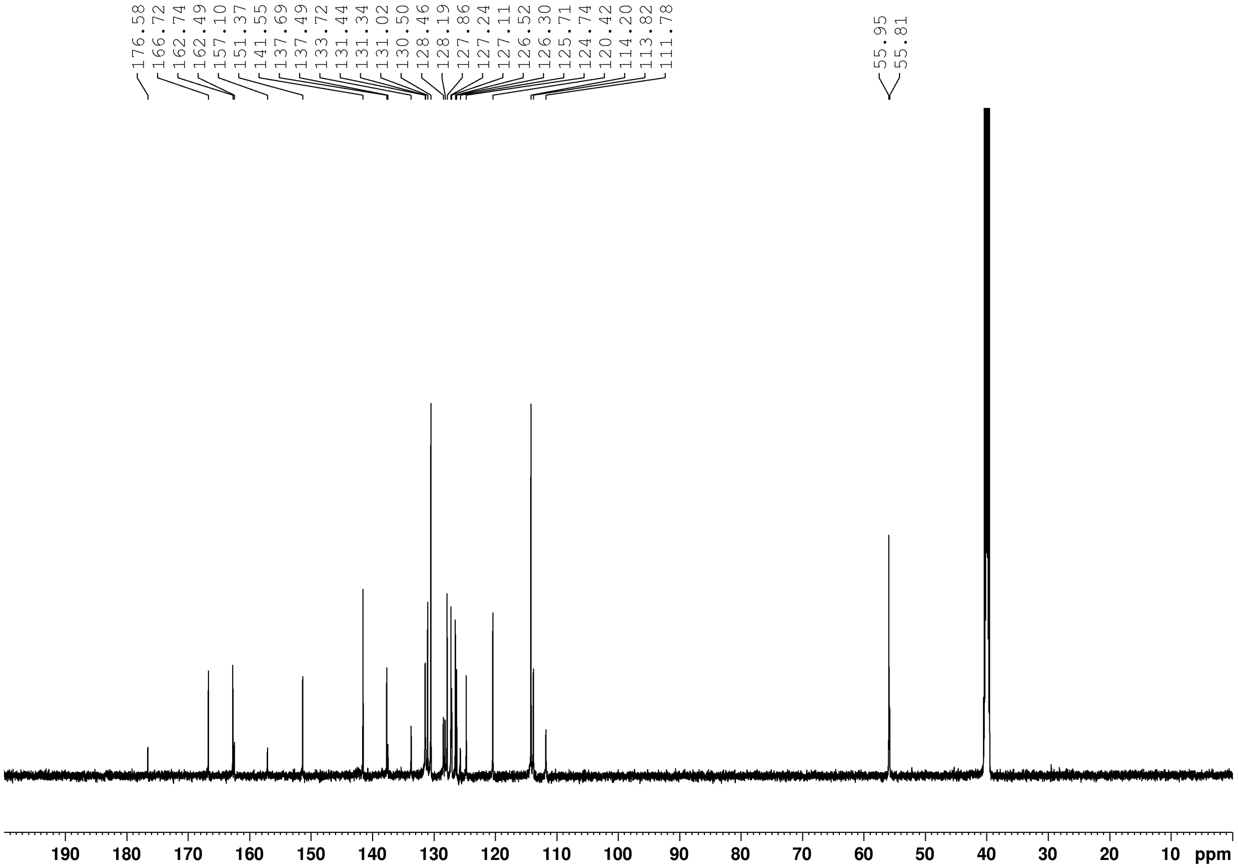

Figure S4: The ^{13}C NMR spectrum of compound **2**

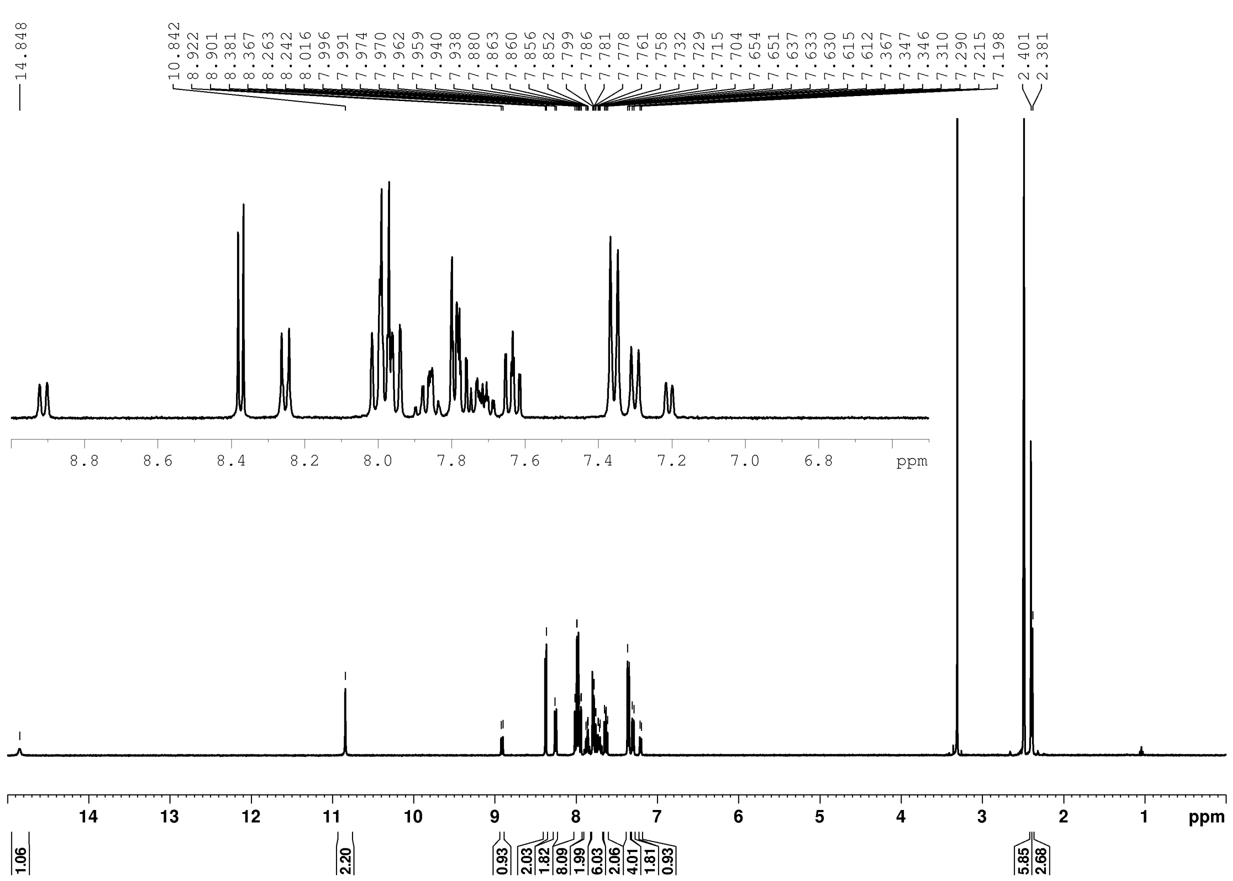

Figure S5: The ^{1}H NMR spectrum of compound **3**

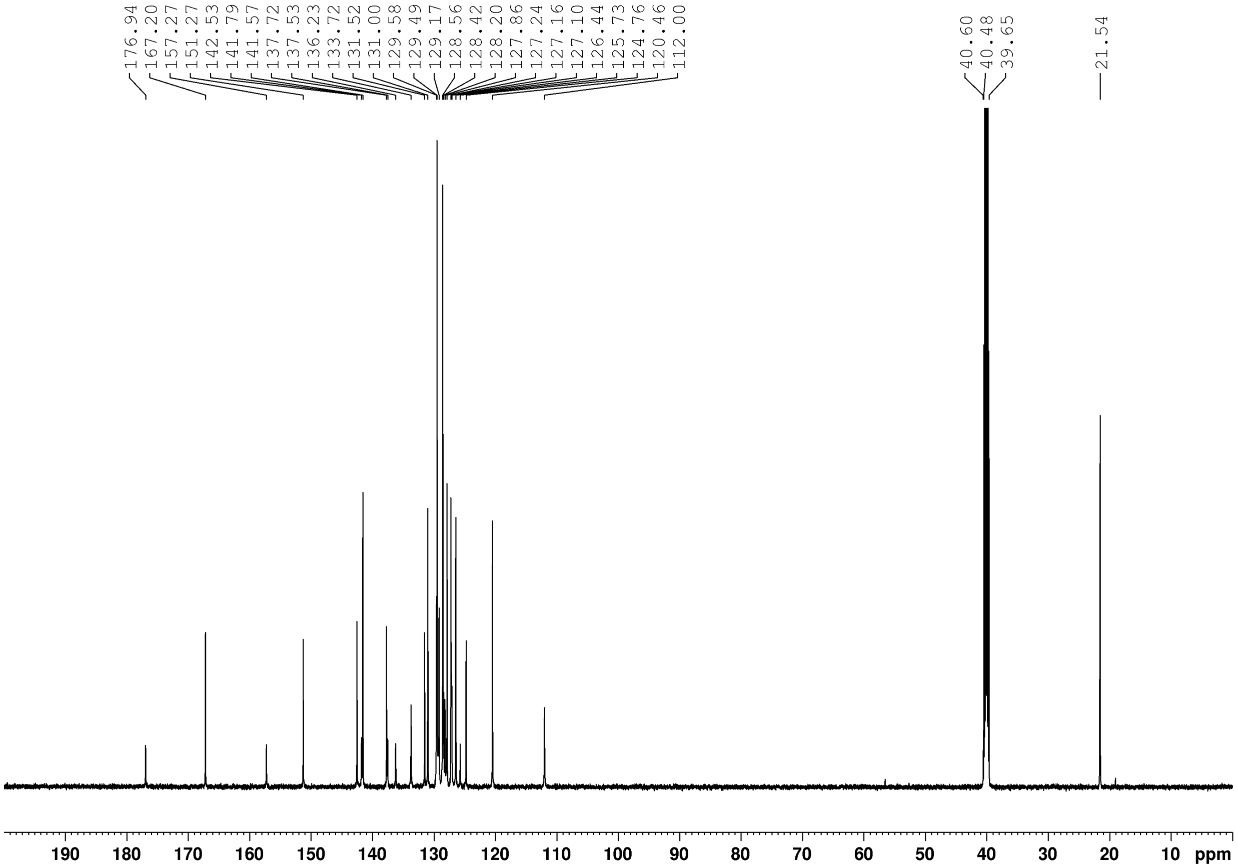

Figure S6: The ^{13}C NMR spectrum of compound **3**

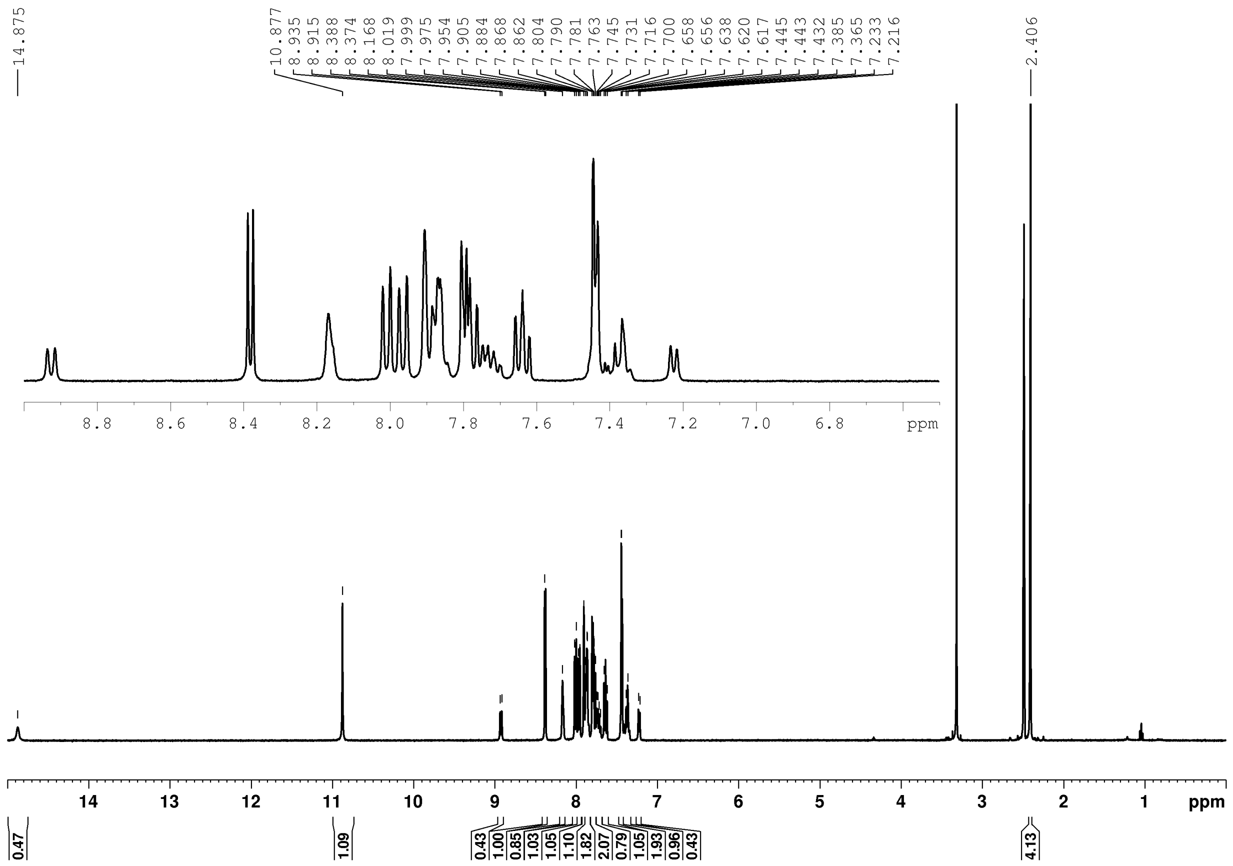

Figure S7: The ^{1}H NMR spectrum of compound **4**

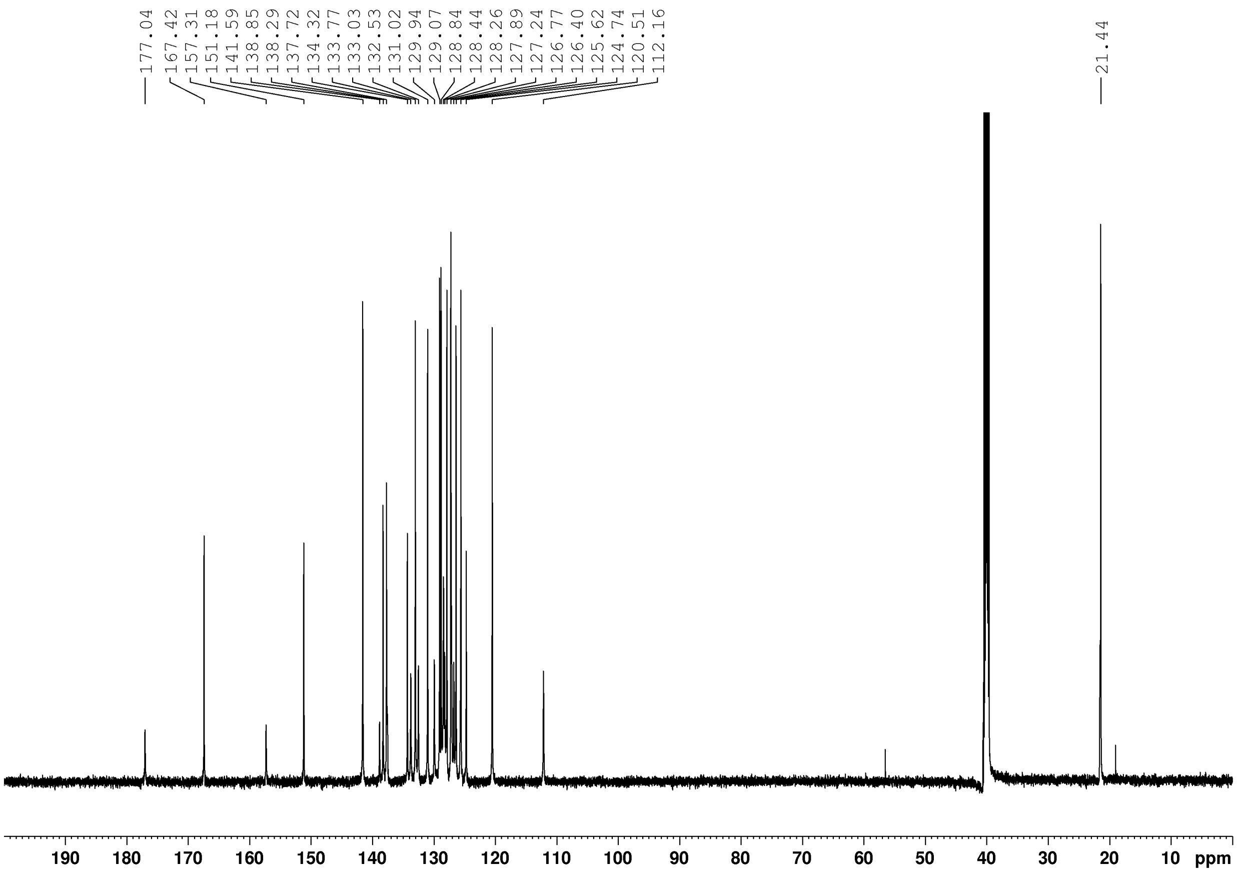

Figure S8: The ^{13}C NMR spectrum of compound **4**

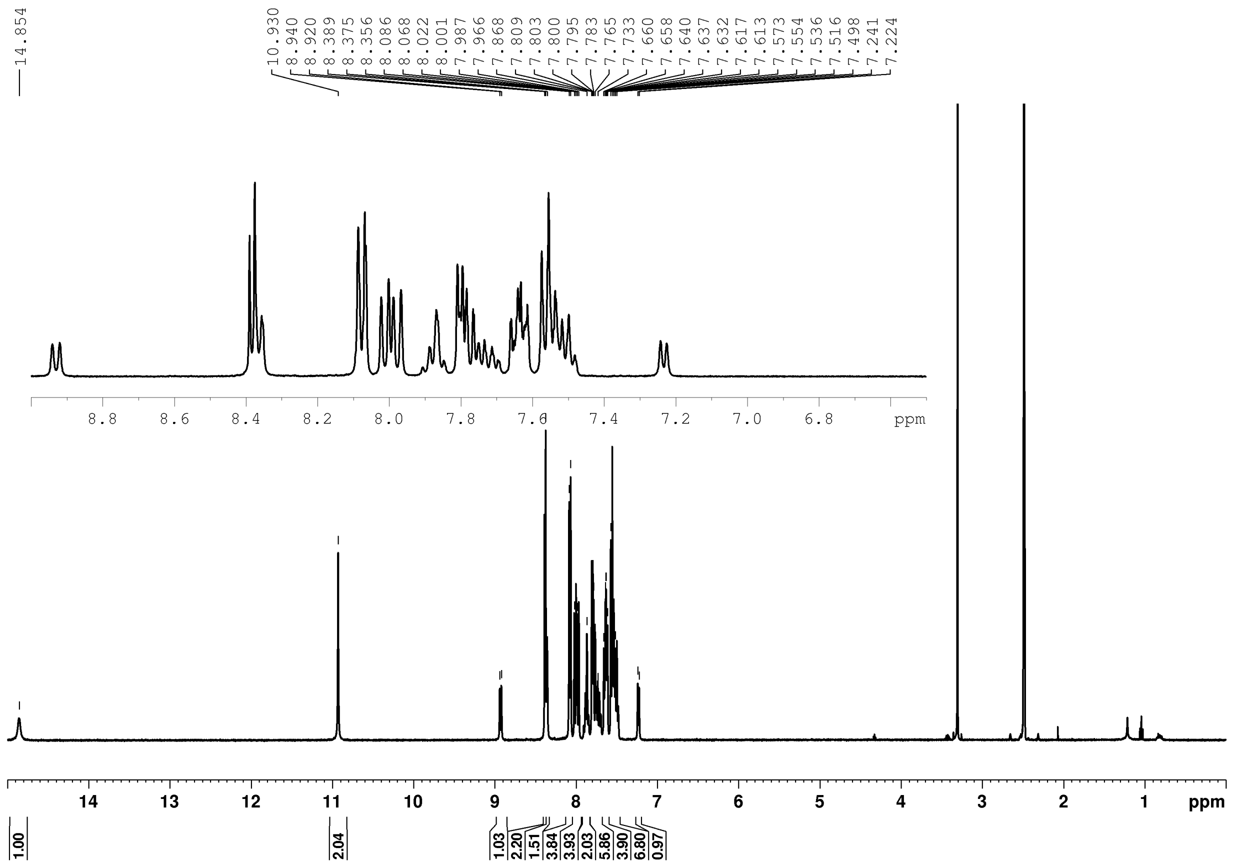

Figure S9: The ^{1}H NMR spectrum of compound **5**

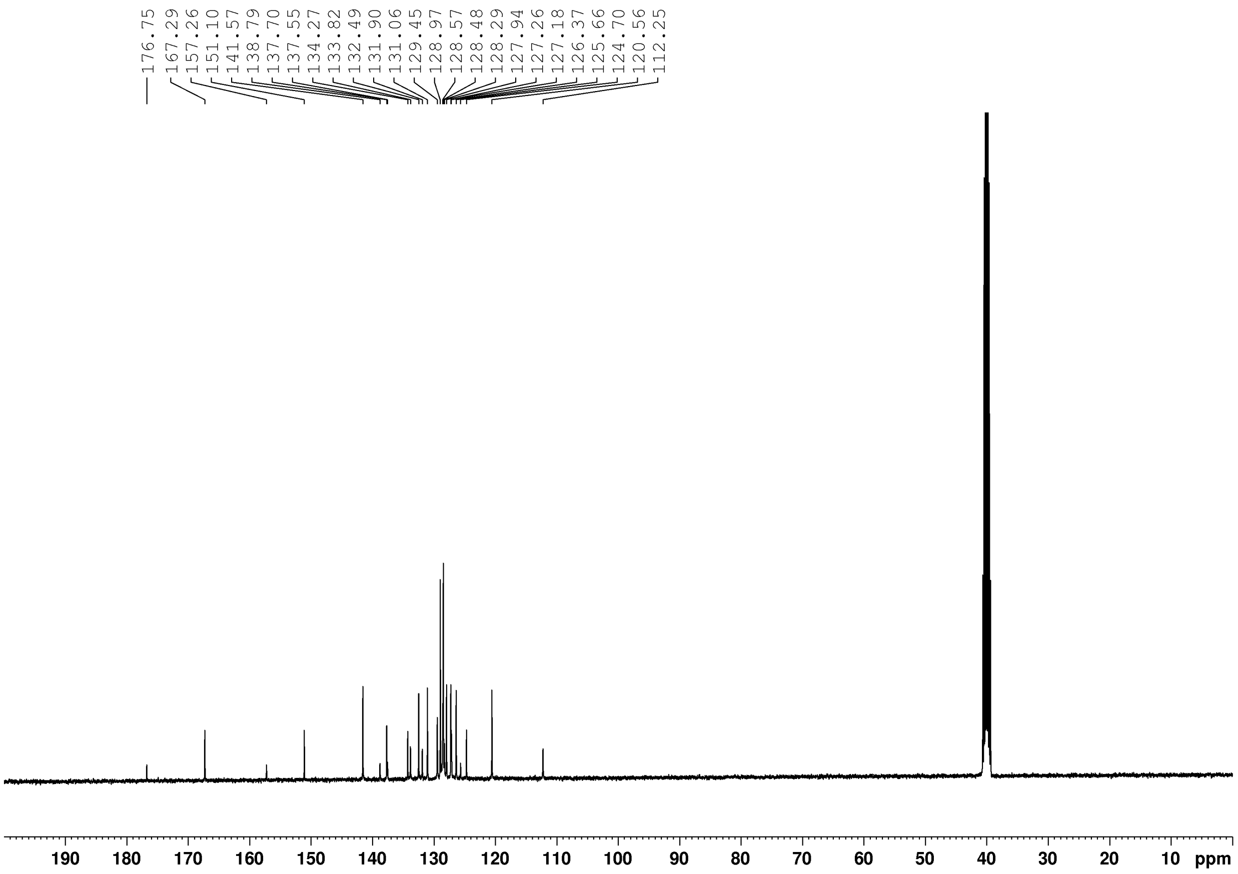

Figure S10: The ^{13}C NMR spectrum of compound **5**

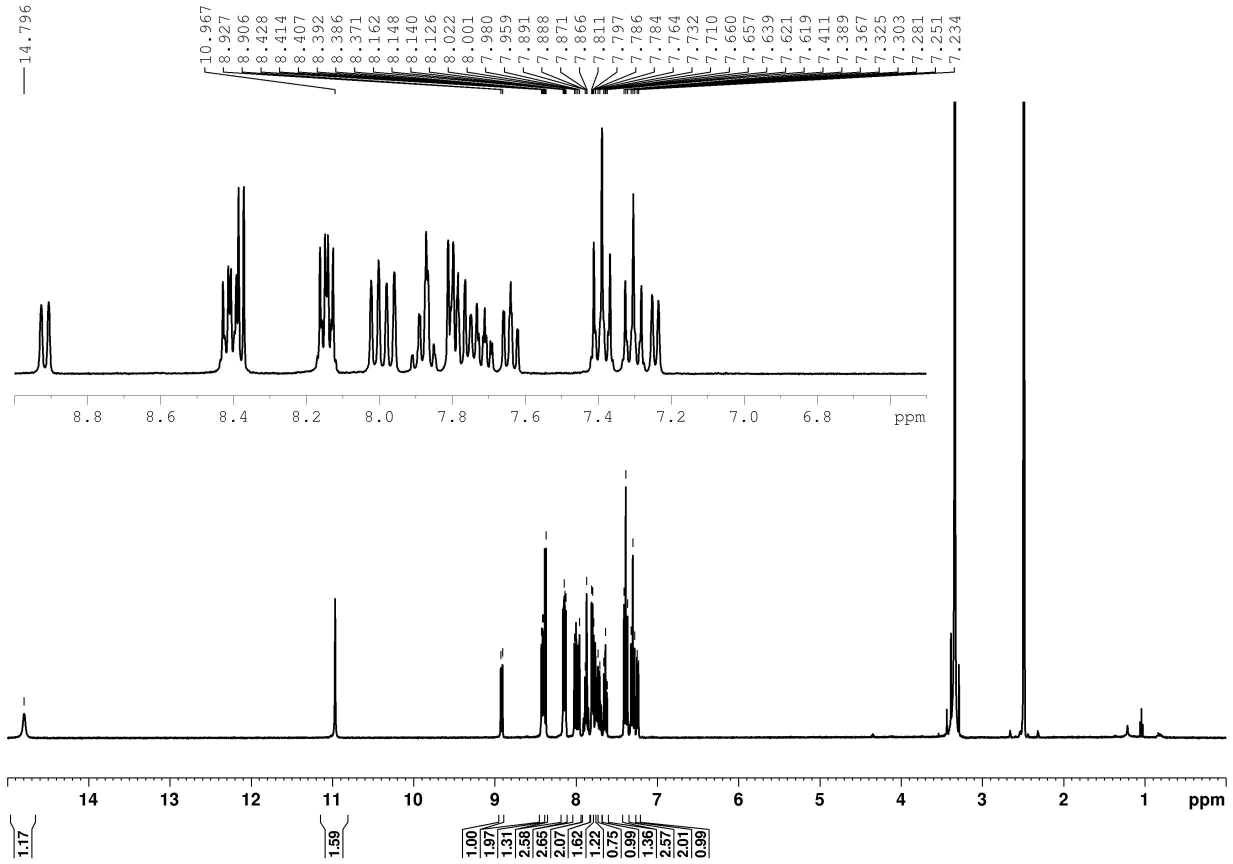

Figure S11: The ^{1}H NMR spectrum of compound **6**

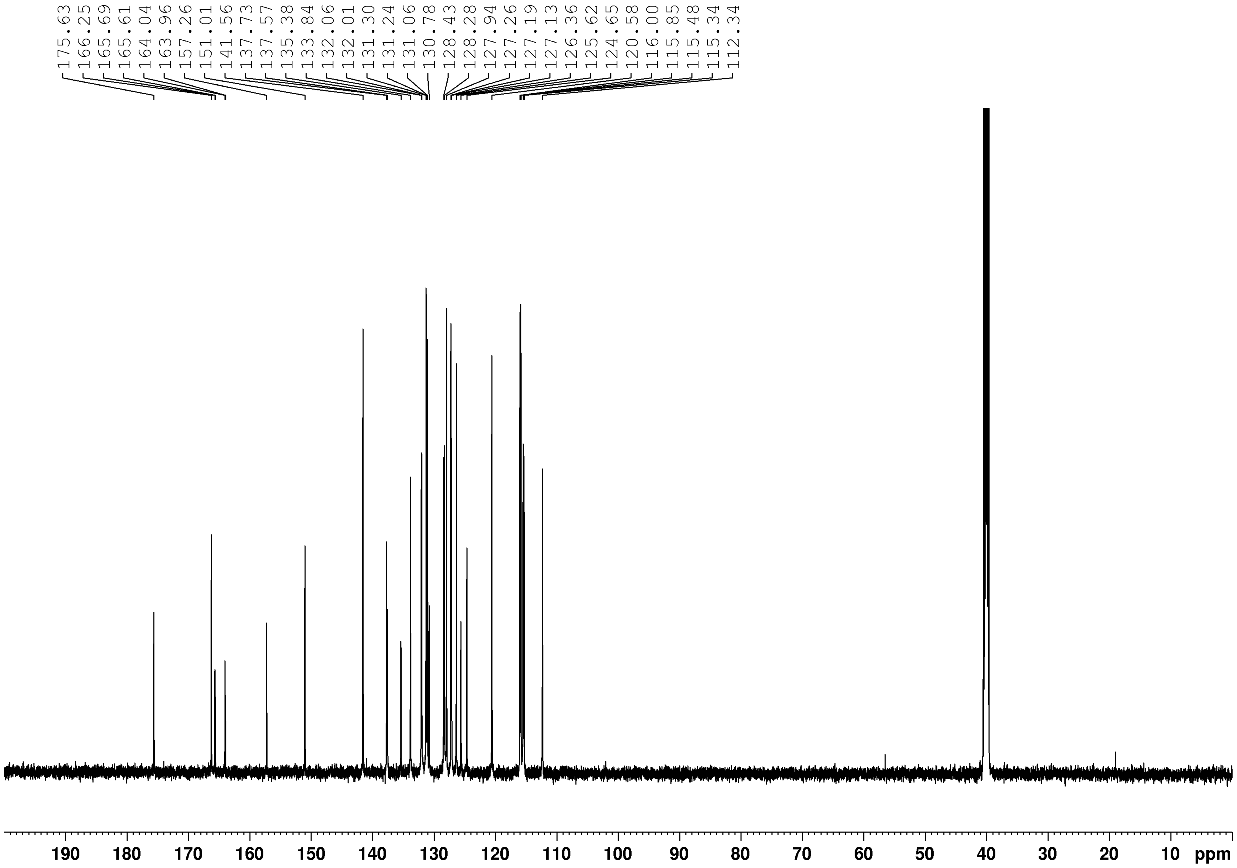

Figure S12: The ^{13}C NMR spectrum of compound **6**

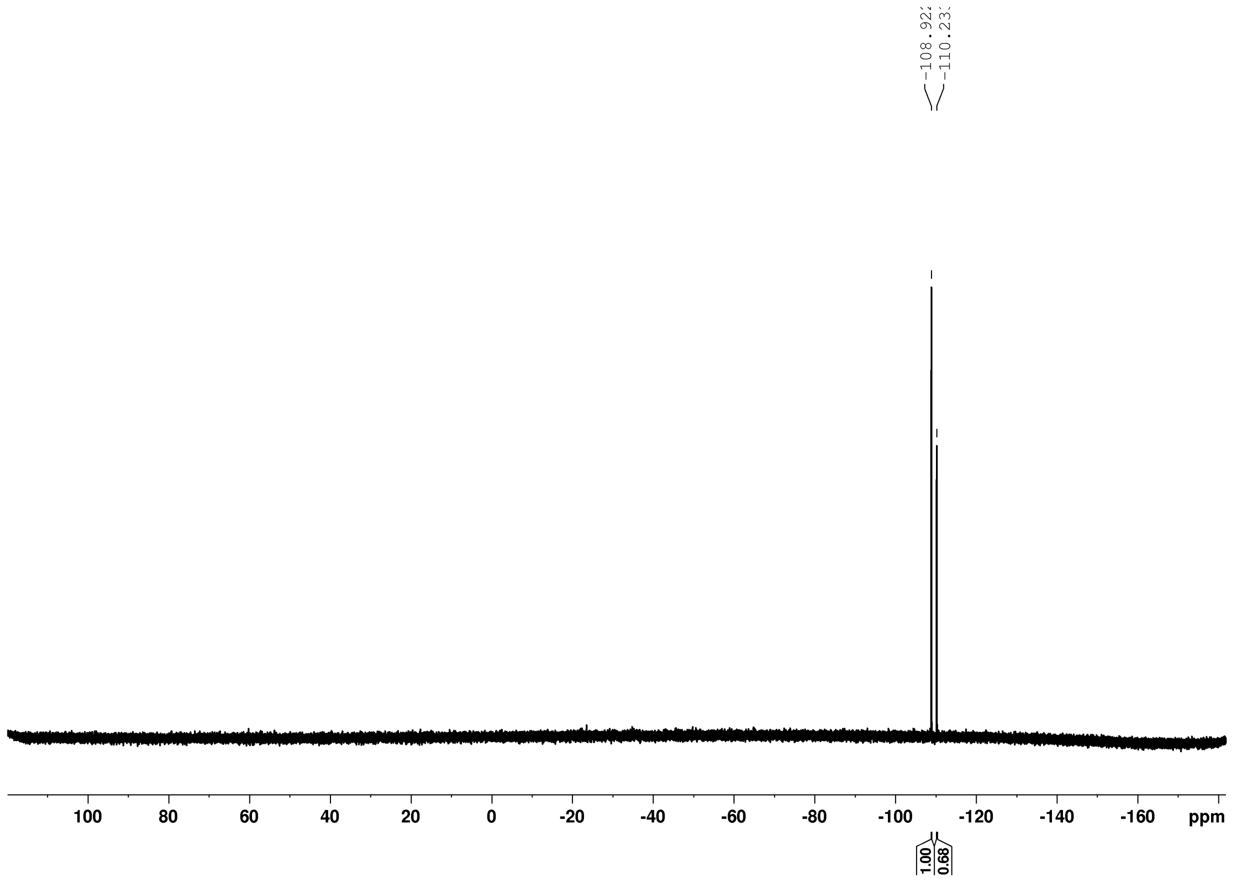

Figure S13: The ^{19}F NMR spectrum of compound **6**

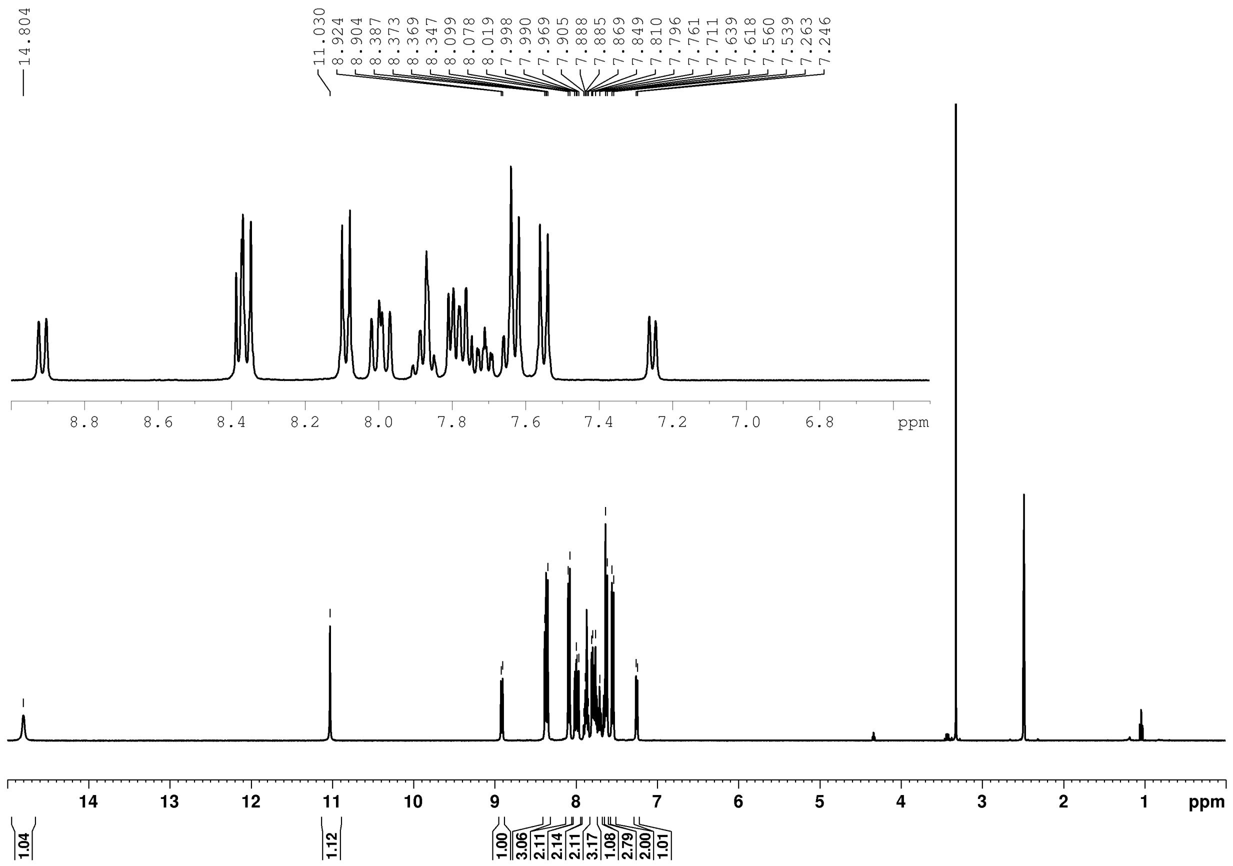

Figure S14: The ^{1}H NMR spectrum of compound **7**

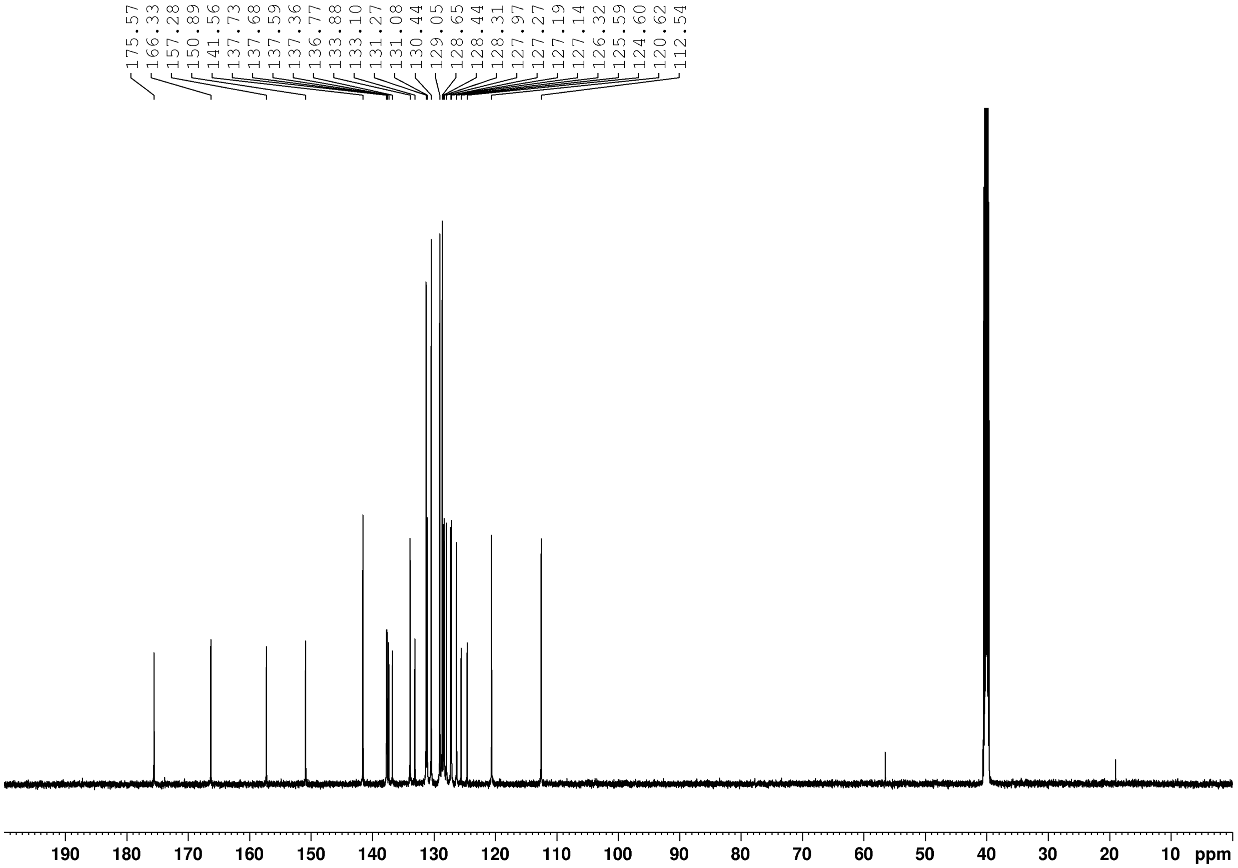

Figure S15: The ^{13}C NMR spectrum of compound **7**

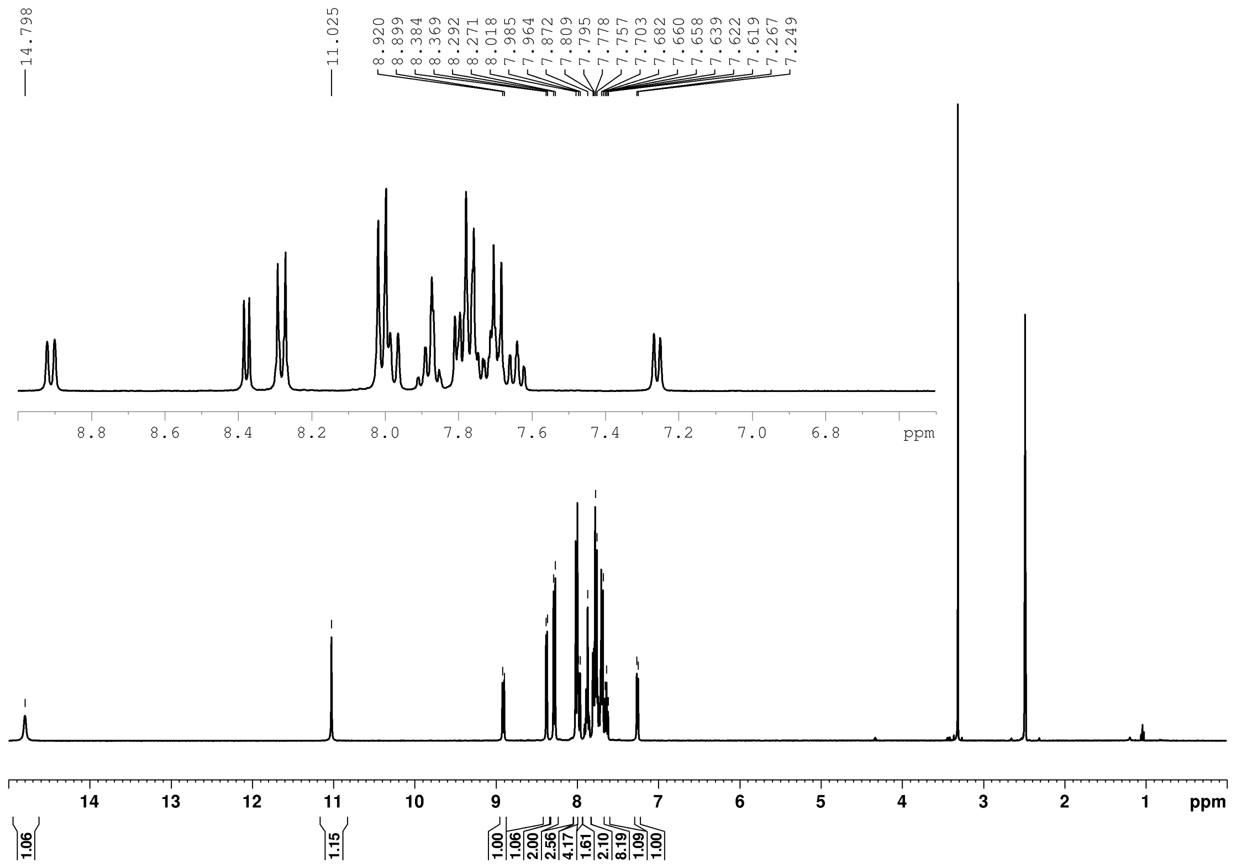

Figure S16: The ^{1}H NMR spectrum of compound **8**

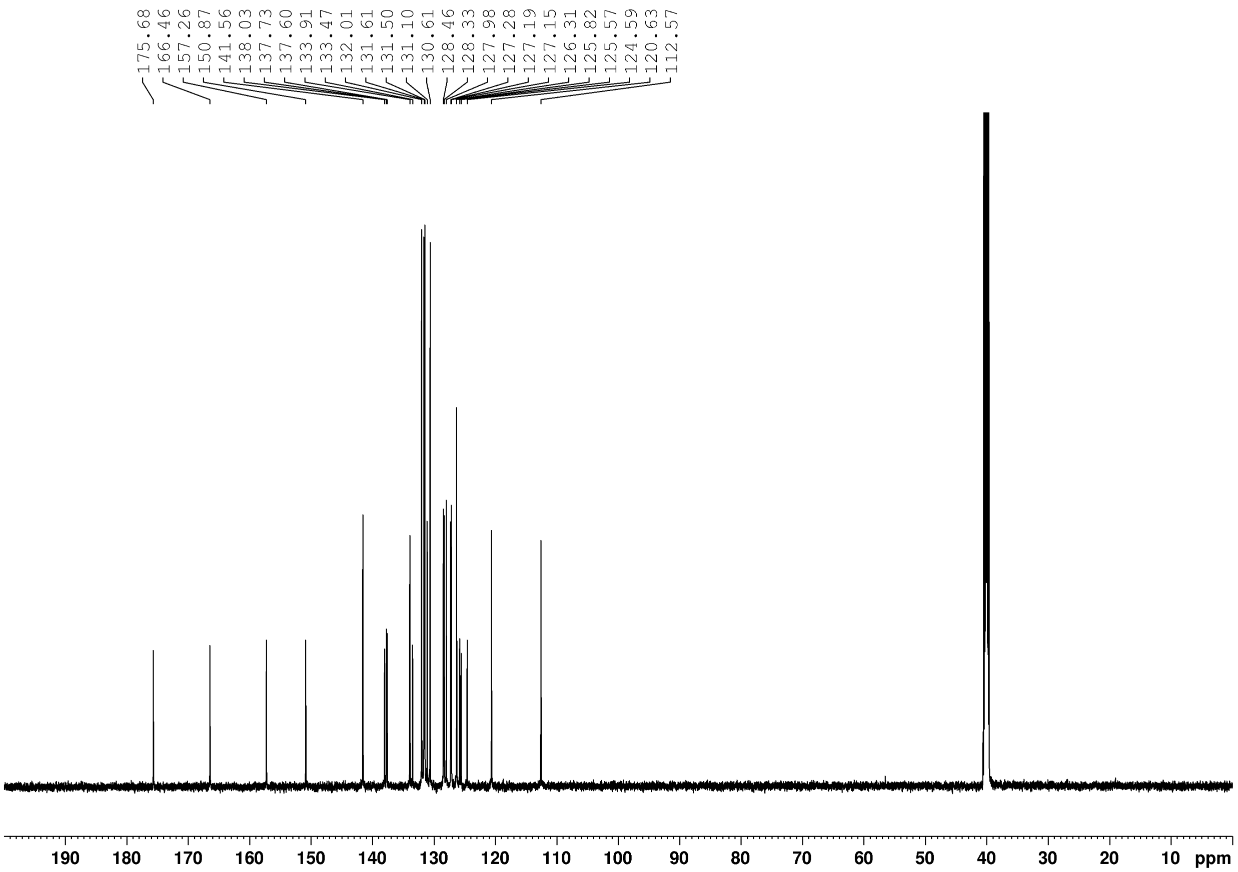

Figure S17: The ^{13}C NMR spectrum of compound **8**

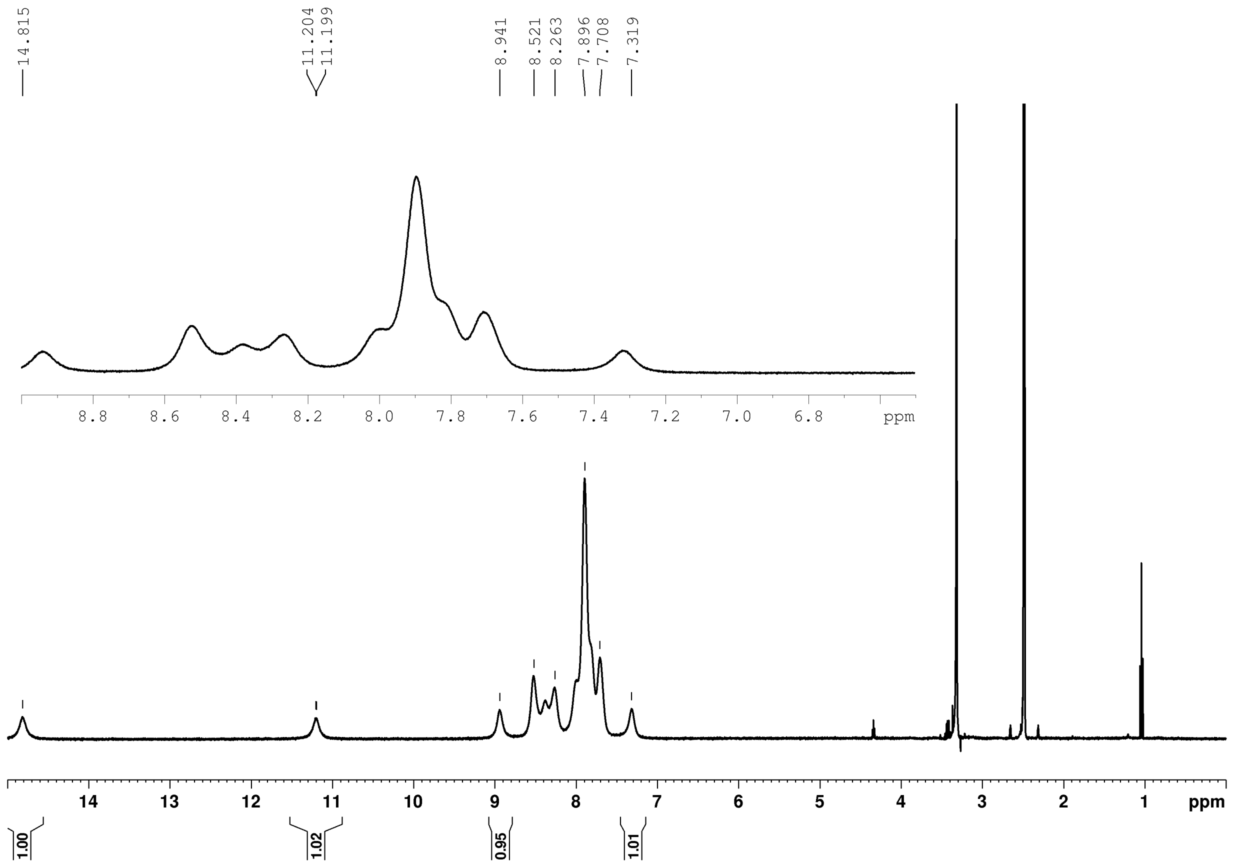

Figure S18: The ^{1}H NMR spectrum of compound **9**

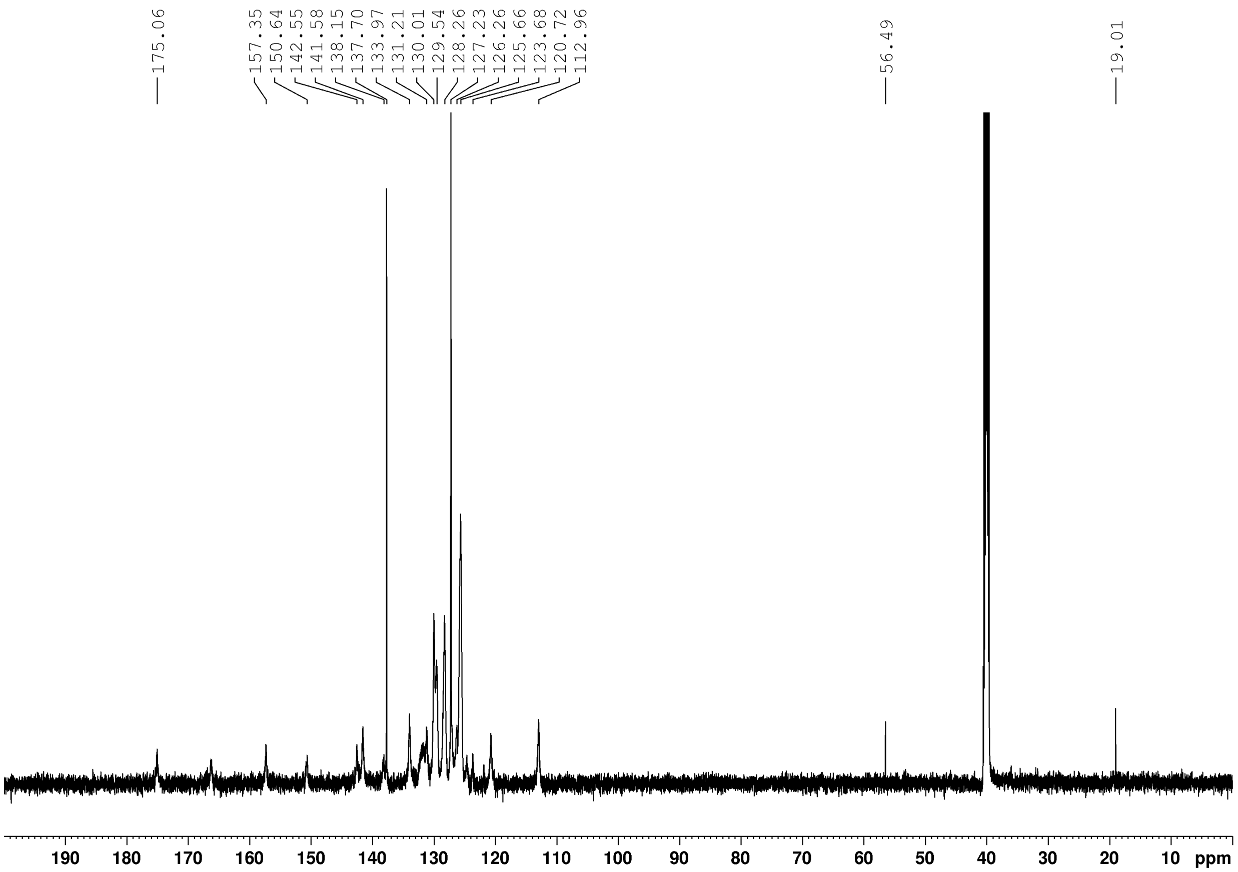

Figure S19: The ^{13}C NMR spectrum of compound **9**

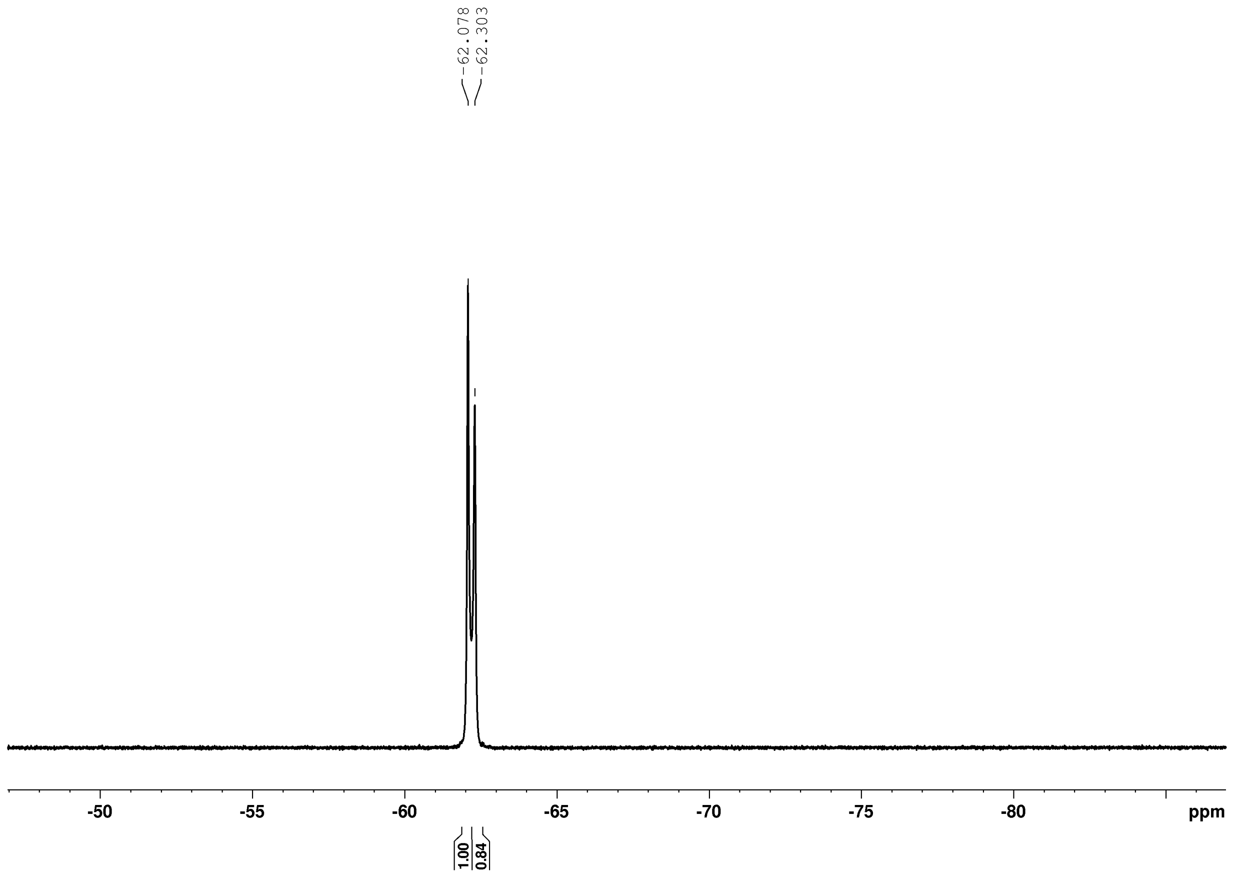

Figure S20: The ^{19}F NMR spectrum of compound **9**

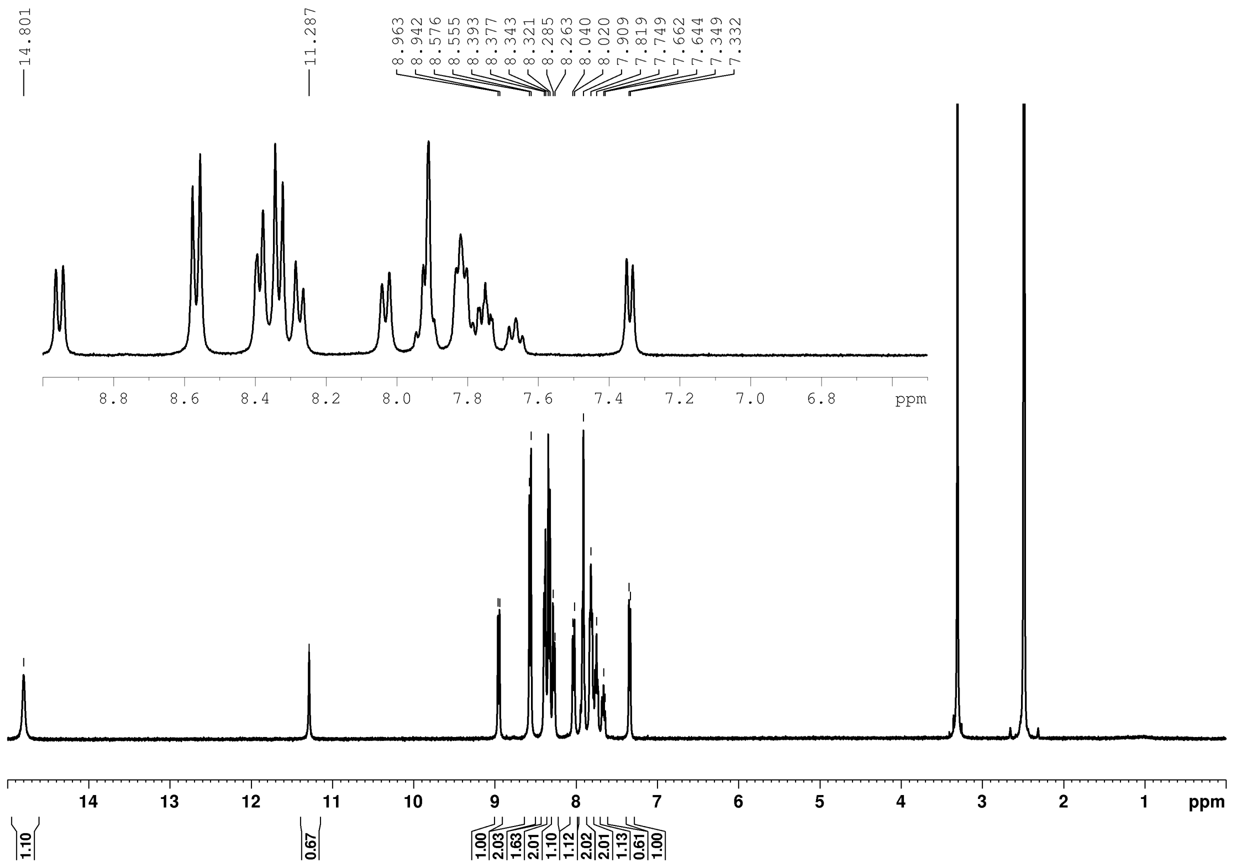

Figure S21: The ^{1}H NMR spectrum of compound **9**

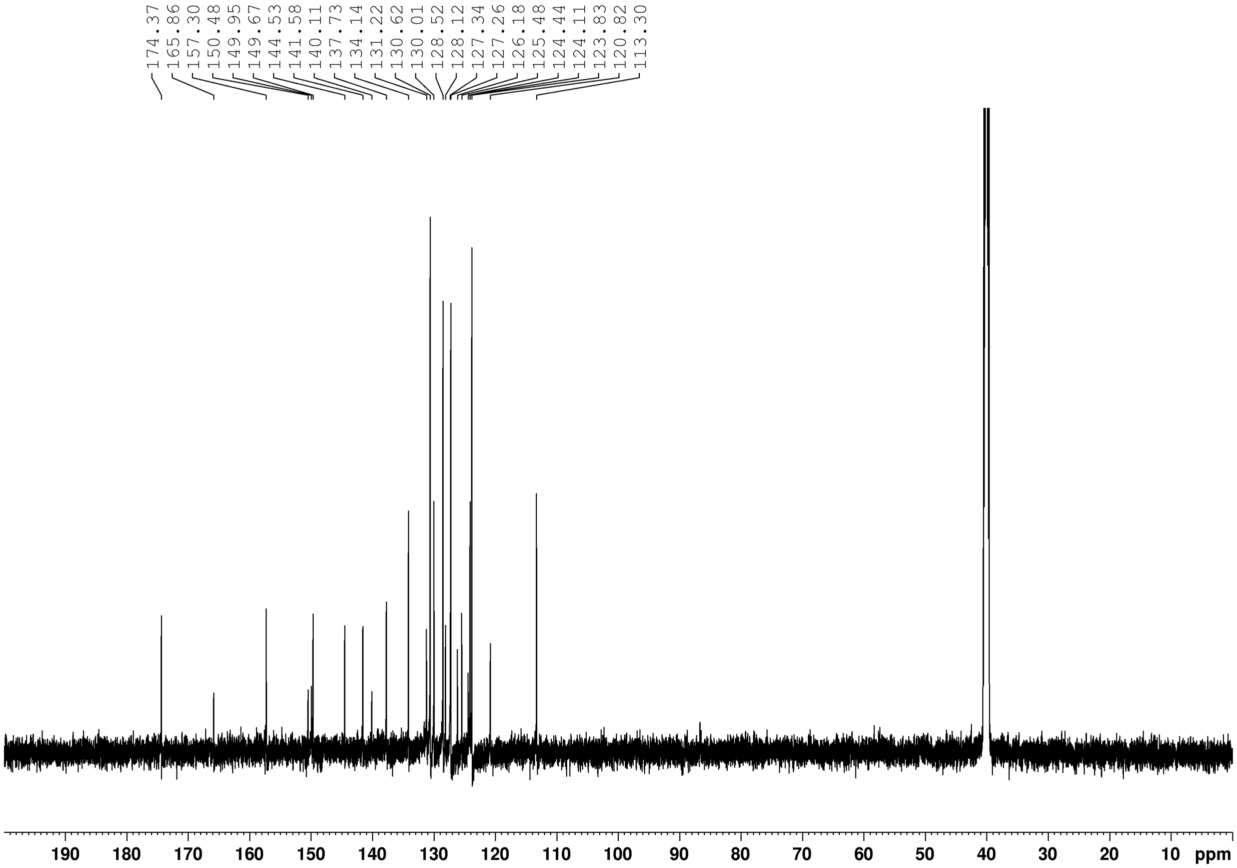

Figure S22: The ^{13}C NMR spectrum of compound **9**

Theoretical calculations

Figs. S23-S25 present the lowest-energy (obtained from CREST calculations) ωB97X-D/def2-TZVP reoptimized forms of **1**, **5** and **10** in implicit PCM solvent (DMSO). One should notice the conformational preferences for the amide forms: the most stable reoptimized amide adapts the unfolded form, while the correlation-including methods such as MP2 clearly prefer the folded amide with the direct dispersion interaction of the two aromatic parts of the molecule. Such an overestimation of dispersion energy exclude MP2 approach and double-hybrid DFT functionals from the present considerations.

Table S1: Relative Gibbs free energies for **1**, **5** and **10** tautomers in various approaches in PCM [kcal/mol] (the DLPNO-CCSD(T) corrected for solvent effects and zero-point energy with the DFT contributions)

System	A	E	O
	ωB97X-D/def2TZVP/PCM		
**1** (4-NMe_2)	1.10	0.00	5.32
**5** (H)	1.83	0.00	6.34
**10** (4-NO_2)	2.56	0.00	6.35
	CBS-QB3		
**1** (4-NMe_2)	0.45	0.00	3.53
**5** (H)	0.72	0.00	3.73
**10** (4-NO_2)	1.73	0.00	3.96
	DLPNO-CCSD(T)/def2TZVPP		
**1** (4-NMe_2)	0.00	2.57	7.43
**5** (H)	0.00	2.23	7.31
**10** (4-NO_2)	0.00	1.57	6.86

NMR chemical shifts from DFT calculations

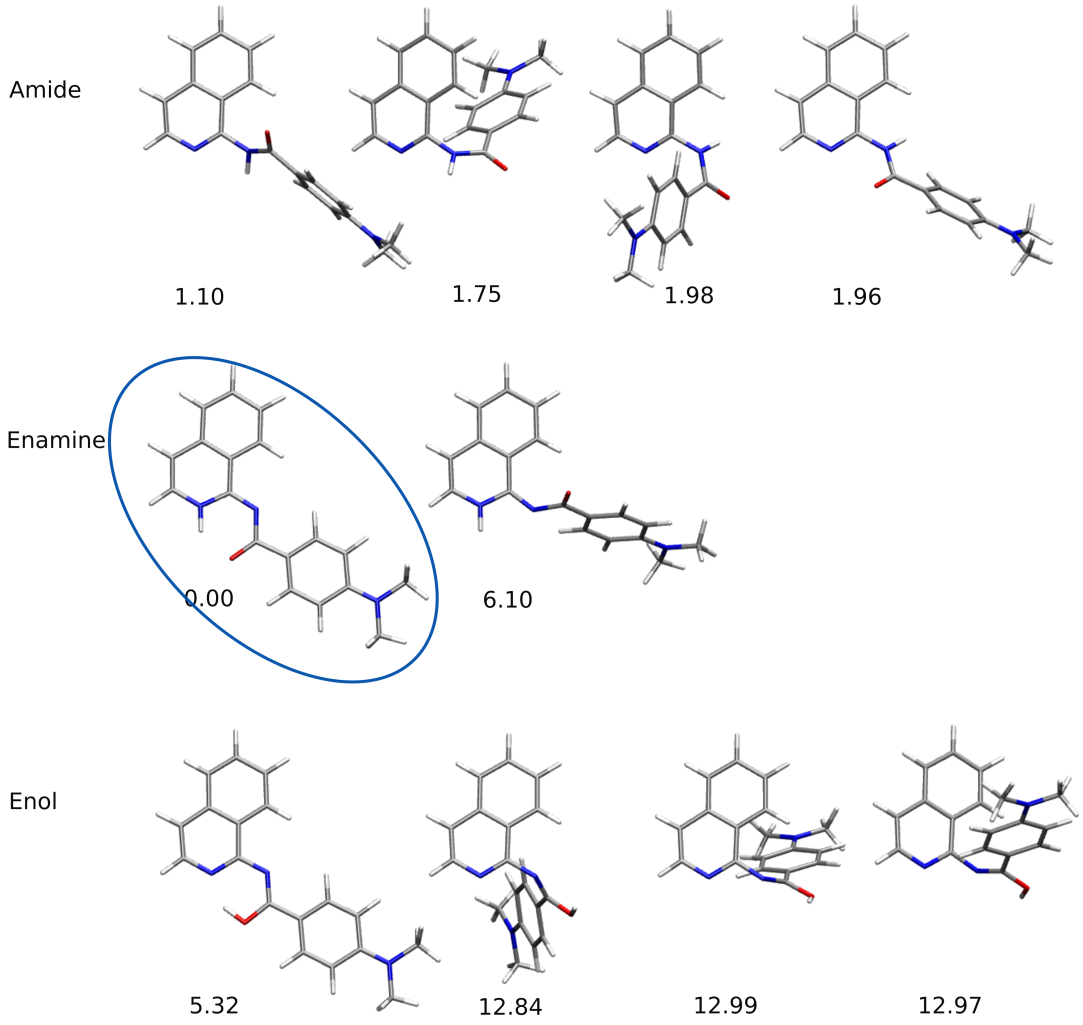

Figure S23: The reoptimized lowest energy structures of the **1** with implicit PCM solvent model (the relative Gibbs free energies in kcal/mol; the lowest energy tautomer in the blue circle)

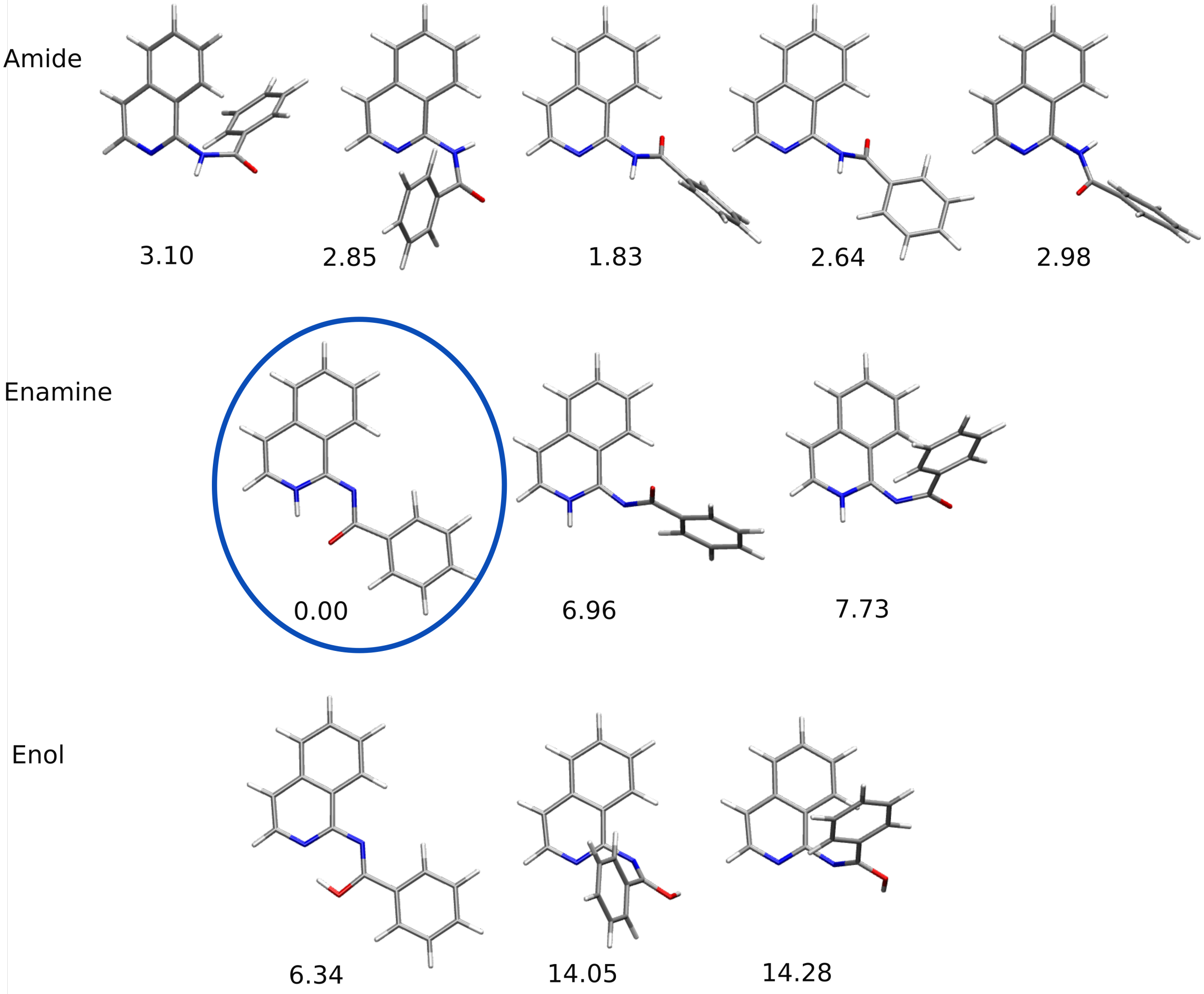

Figure S24: The reoptimized lowest energy structures of the **5** with implicit PCM solvent model (the relative Gibbs free energies in kcal/mol; the lowest energy tautomer in the blue circle)

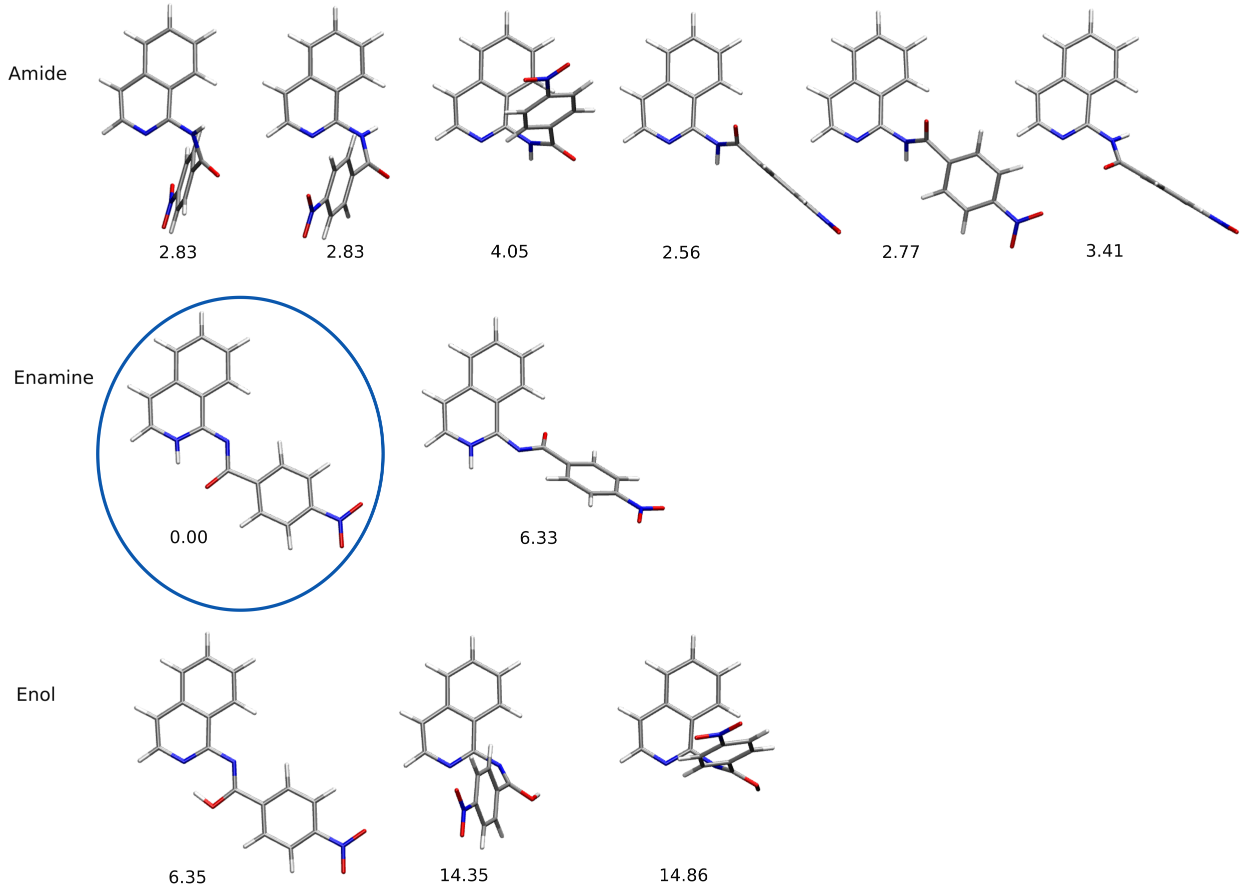

Figure S25: The reoptimized lowest energy structures of the **10** with implicit PCM solvent model (the relative Gibbs free energies in kcal/mol; the lowest energy tautomer in the blue circle)

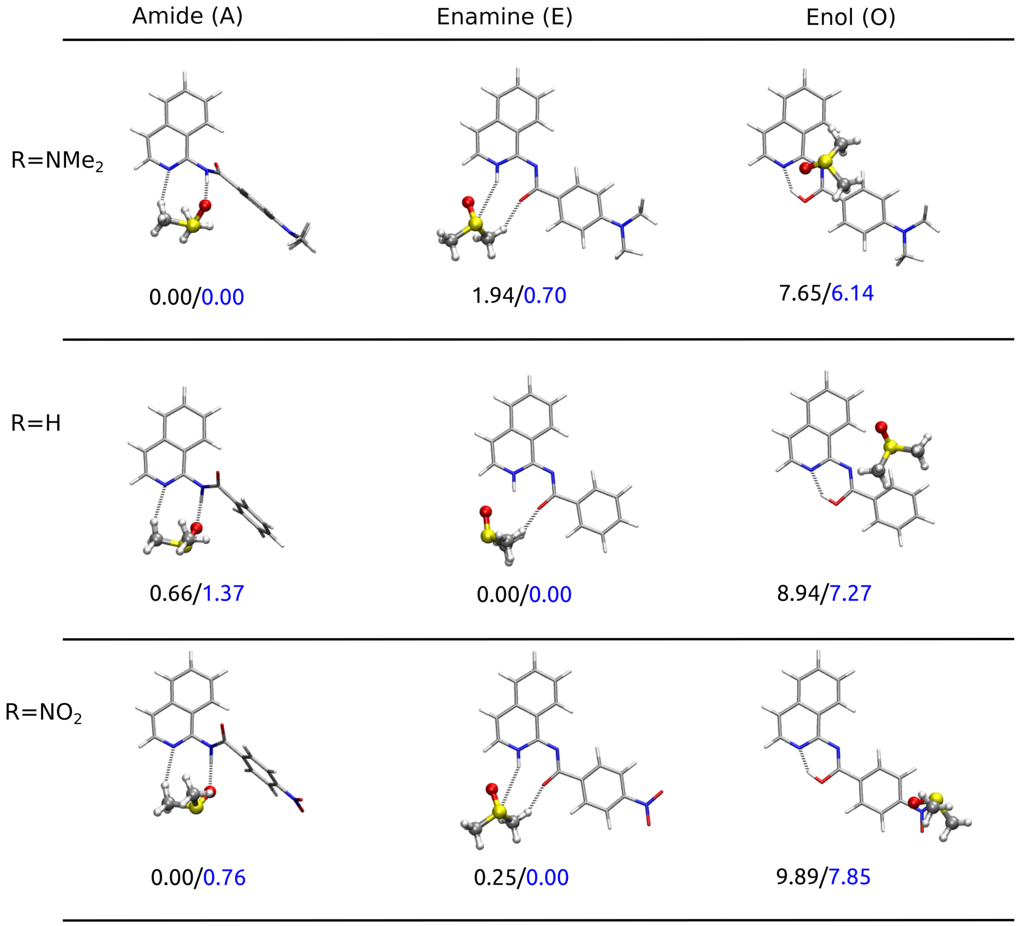

Figure S26: The optimized solute:solvent complexes in vacuum together with the relative energy ΔE (in black) and the relative Gibbs free energy ΔG (in blue) [kcal/mol]

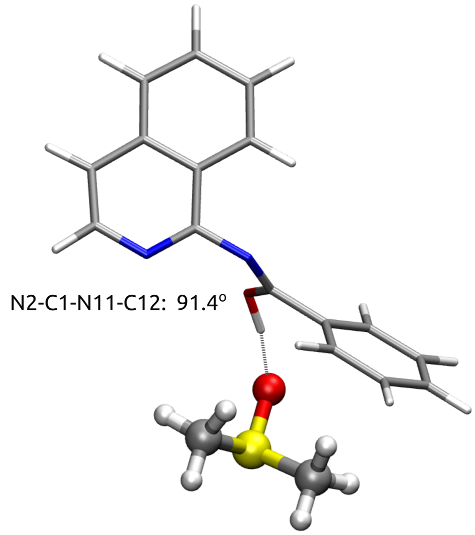

Figure S27: Optimized **5** enol tautomer with the twisted structure interacting with the explicit DMSO molecule

Table S2: Distances in the quasi-ring formed by the intramolecular hydrogen bonds for **1**, **5** and **10** tautomers in ωB97X-D/def2TZVP/PCM [Å]

System	Enamine			Enol		
	N2–H	H...O13	N2...O13	N2...H	H–O13	N2...O13
**1** (4-NMe_2)	1.0227	1.7662	2.5813	1.5796	1.0170	2.5001
**5** (4-H)	1.0222	1.7722	2.5852	1.5752	1.0196	2.4967
**10** (4-NO_2)	1.0221	1.7819	2.5922	1.5724	1.0217	2.4943

Table S3: Chemical shifts δ in ppm for ^{1}H NMR ωB97X-D/def2-TZVP

	Experiment		Implicit solvent			Explicit solvent			Explicit-implicit solvent		
Compound	A(H11)	E(H2)	A	E	O	A	E	O	A	E	O
**1** (4-NMe_2)	10.53	14.87	8.11	15.10	18.35	11.21	14.43	18.10	11.48	14.46	18.09
**2** (4-OMe)	10.77	14.86	8.16	15.08	18.59	11.37	14.56	17.96	11.68	14.63	18.52
**3** (4-Me)	10.84	14.89	8.22	15.15	18.56	11.48	14.61	17.94	11.74	14.63	18.20
**4** (3-Me)	10.88	14.98	8.23	15.10	18.56	11.34	14.49	17.84	11.79	12.98	18.31
**5** (4-H)	10.96	14.83	8.25	15.15	18.73	11.60	14.62	16.84	11.86	14.69	18.30
**6** (4-F)	10.98	14.80	8.19	15.12	18.70	11.58	14.46	18.71	11.81	14.46	18.69
**7** (4-Cl)	11.03	14.83	8.20	15.06	18.72	11.64	14.47	18.75	11.91	14.46	18.71
**8** (4-Br)	11.03	14.83	8.20	15.08	18.80	11.64	14.46	17.60	11.89	14.48	18.49
**9** (4-CF_3)	11.20	14.85	8.27	15.09	18.86	11.81	14.51	18.13	12.06	14.53	18.80
**10** (4-NO_2)	11.29	14.82	8.26	15.08	18.96	11.95	14.51	18.15	12.16	14.53	18.88

Non-covalent interactions

Table S4: AIM parameters: electron density ρ, its laplacian $\nabla^2\rho$, total energy density H_b[S1] in a. u. for the indicated bond critical points and hydrogen-bond energy $E(HB)$ in kcal/mol, according to Espinosa,[S2] estimated in the implicit solvent model within the ωB97X-D/def2-TZVP approach for enamine and enol tautomers

	Enamine (O13$\cdots$H2)				Enol (N2$\cdots$H13)			
Compound	ρ	$\nabla^2\rho$	H_b	E(HB)	ρ	$\nabla^2\rho$	H_b	E(HB)
**1** (4-NMe_2)	0.0434	0.1375	-0.0047	-13.72	0.0731	0.1085	-0.0242	-23.71
**2** (4-OMe)	0.0428	0.1369	-0.0044	-13.49	0.0738	0.1074	-0.0248	-23.96
**3** (4-Me)	0.0429	0.1372	-0.0044	-13.54	0.0732	0.1078	-0.0243	-23.72
**4** (3-Me)	0.0425	0.1365	-0.0042	-13.35	0.0731	0.1078	-0.0242	-23.67
**5** (4-H)	0.0427	0.1369	-0.0043	-13.46	0.0740	0.1070	-0.0249	-24.03
**6** (4-F)	0.0427	0.1370	-0.0043	-13.46	0.0740	0.1068	-0.0249	-24.01
**7** (4-Cl)	0.0422	0.1360	-0.0041	-13.22	0.0740	0.1066	-0.0249	-23.98
**8** (4-Br)	0.0423	0.1362	-0.0041	-13.26	0.0744	0.1062	-0.0252	-24.16
**9** (4-CF_3)	0.0420	0.1358	-0.0040	-13.15	0.0743	0.1062	-0.0251	-24.11
**10** (4-NO_2)	0.0417	0.1354	-0.0038	-13.03	0.0747	0.1053	-0.0254	-24.23

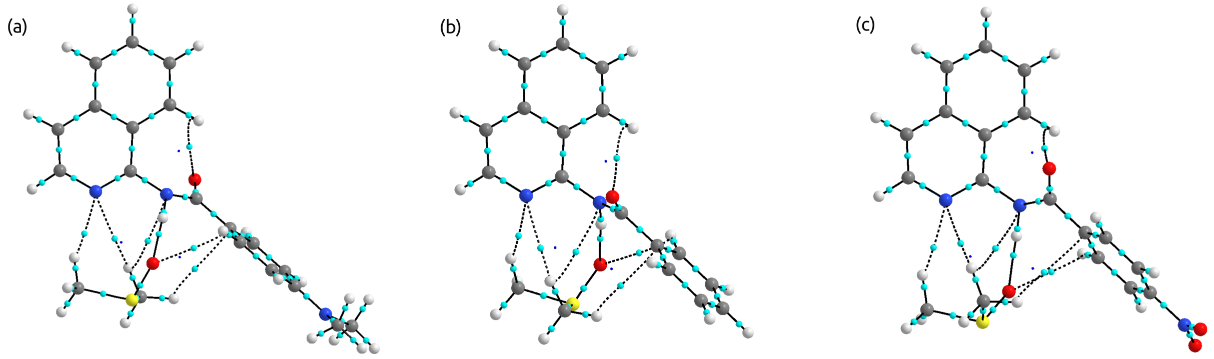

Figure S28: Molecular graphs explicit interaction of (a) **1**, (b) **5** and (c) **10** amide tautomer with DMSO molecule

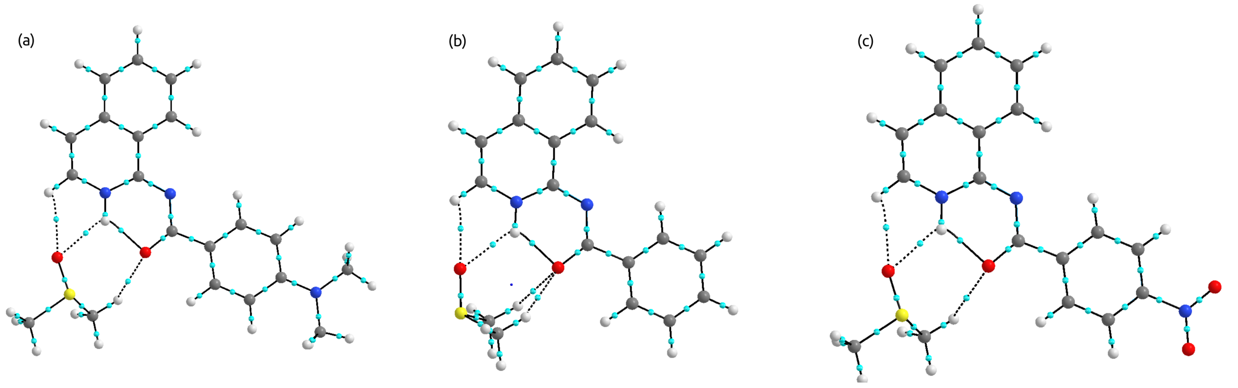

Figure S29: Molecular graphs explicit interaction of (a) **1**, (b) **5** and (c) **10** enamine tautomer with DMSO molecule

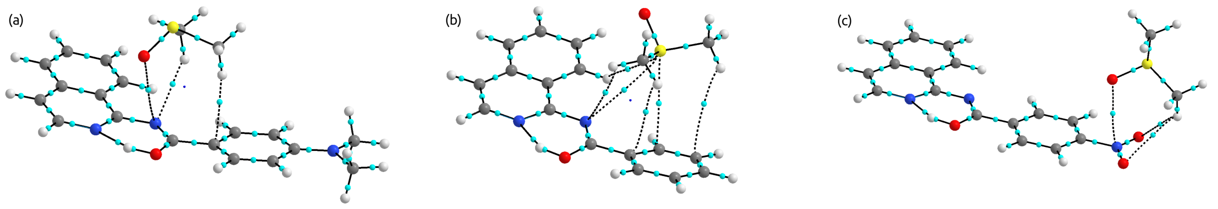

Figure S30: Molecular graphs explicit interaction of (a) **1**, (b) **5** and (c) **10** enole tautomer with DMSO molecule

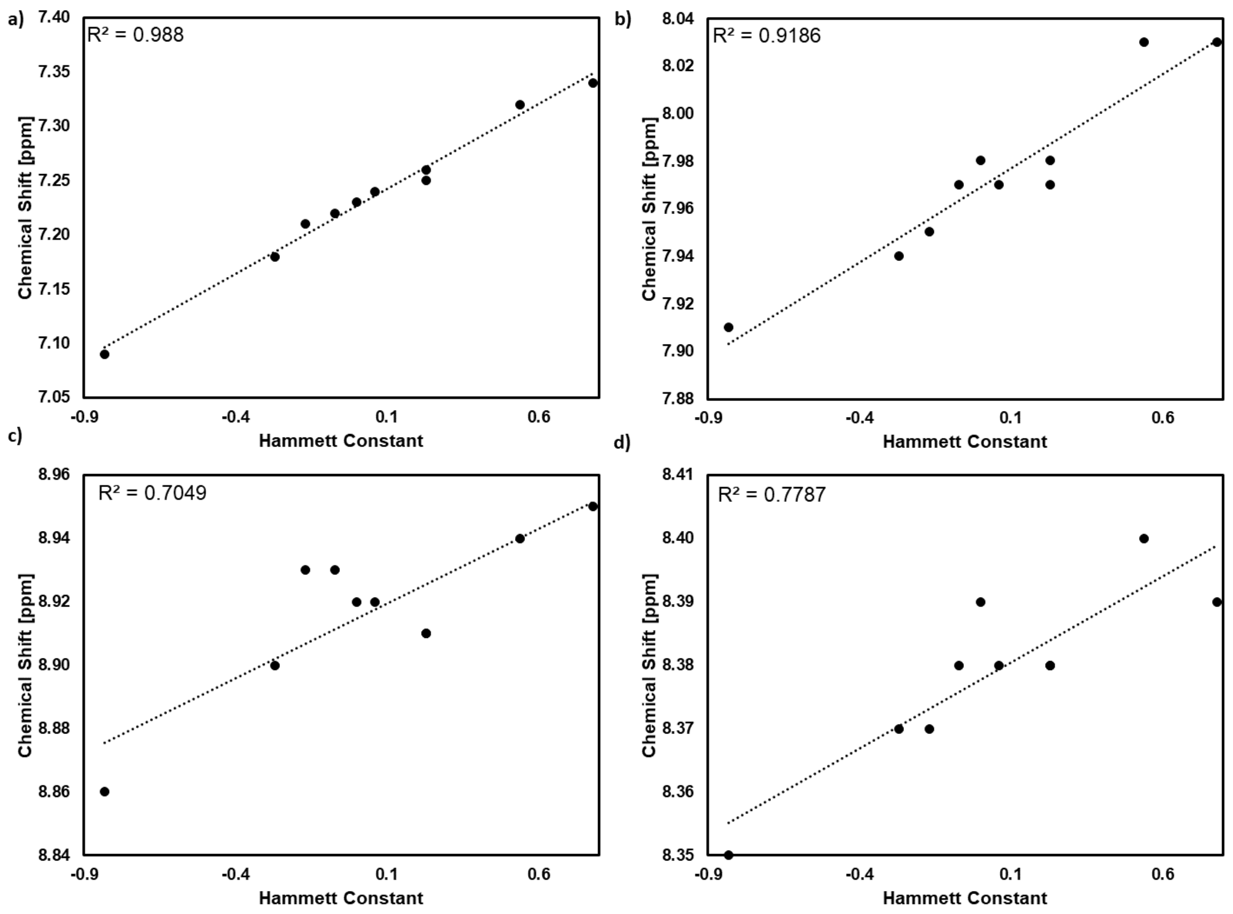

Figure S31: Correlation between Hammett substituent constant and ^{1}H NMR shifts of protons H9 (form **E** - a and **A** - b) and H3 (form **E** - c and **A** - d)

Table S5: AIM parameters: electron density ρ, its laplacian $\bigtriangledown^2\rho$ and total energy density H_b[S1] in a. u. for selected bond critical points (BCP) and hydrogen-bond energy $E(HB)$ in kcal/mol, according to Espinosa,[S2] estimated in the explicit solvent model within the ωB97X-D/def2-TZVP approach for amide tautomer of **1**, **5** and **10** (H(DMSO:1) and H(DMSO:2) denote the hydrogens from the DMSO methyl groups; compare the molecular graphs in Fig. S28)

Compound	BCP	ρ	$\bigtriangledown^2\rho$	H_b	E(HB)
		**Amide**			
**1** (4-NMe_2)	O13$\cdots$H9 (intramol)	0.0093	0.0400	0.0019	-1.94
	N2$\cdots$H(DMSO:1)	0.0120	0.0363	0.0015	-1.91
	N2$\cdots$H(DMSO:2)	0.0060	0.0185	0.0008	-0.93
	N11$\cdots$H(DMSO:1)	0.0062	0.0236	0.0013	-1.06
	C14$\cdots$H(DMSO:3)	0.0055	0.0167	0.0008	-0.81
	H19$\cdots$O(DMSO)	0.0095	0.0365	0.0016	-1.83
	H11$\cdots$O(DMSO)	0.0285	0.0987	0.0009	-7.21
**5** (4-H)	O13$\cdots$H9 (intramol)	0.0092	0.0396	0.0019	-1.92
	N2$\cdots$H(DMSO:1)	0.0061	0.0186	0.0008	-0.93
	N2$\cdots$H(DMSO:2)	0.0117	0.0354	0.0015	-1.85
	N11$\cdots$H(DMSO:1)	0.0060	0.0228	0.0012	-1.01
	C14$\cdots$H(DMSO:3)	0.0049	0.0154	0.0008	-0.73
	H19$\cdots$O(DMSO)	0.0089	0.0344	0.0016	-1.69
	H11$\cdots$O(DMSO)	0.0307	0.1038	0.0002	-8.03
**10** (4-NO_2)	O13$\cdots$H9 (intramol)	0.0091	0.0393	0.0019	-1.19
	N2$\cdots$H(DMSO:1)	0.0076	0.0226	0.0010	-1.16
	N2$\cdots$H(DMSO:2)	0.0111	0.0336	0.0014	-1.76
	N11$\cdots$H(DMSO:1)	0.0057	0.0219	0.0012	-0.96
	H19$\cdots$O(DMSO)	0.0092	0.0362	0.0017	-1.79
	C14$\cdots$H(DMSO:3)	0.0036	0.0115	0.0007	-0.49
	H11$\cdots$O(DMSO)	0.0328	0.1085	-0.0006	-8.86

Table S6: AIM parameters: electron density ρ, its laplacian $\nabla^2\rho$ and total energy density H_b[S1] in a. u. for selected bond critical points (BCP) and hydrogen-bond energy $E(HB)$ in kcal/mol, according to Espinosa,[S2] estimated in the explicit solvent model within the ωB97X-D/def2-TZVP approach for enamine and enamine tautomers of **1**, **5** and **10** (H(DMSO:1) and H(DMSO:2) denote the hydrogens from the DMSO methyl groups; compare the molecular graphs in Figs. S29 and S30)

Compound	BCP	ρ	$\nabla^2\rho$	H_b	E(HB)
		**Enamine**			
**1** (4-NMe_2)	H2···O(DMSO)	0.0152	0.0636	0.0029	-3.20
	H3···O(DMSO)	0.0106	0.0452	0.0021	-2.23
	O13···H(DMSO:1)	0.0137	0.0560	0.0025	-2.82
**5** (4-H)	H2···O(DMSO)	0.0156	0.0648	0.0028	-3.31
	H3···O(DMSO)	0.0121	0.0526	0.0024	-2.62
	O13···H(DMSO:1)	0.0126	0.0507	0.0024	-2.49
	O13···H(DMSO:2)	0.0126	0.0507	0.0024	-2.50
**10**(4-NO_2)	O(DMSO)···H2	0.0171	0.0710	0.0029	-3.75
	O(DMSO)···H3	0.0109	0.0473	0.0022	-2.34
	H(DMSO:1)···O13	0.0121	0.0496	0.0023	-2.44
		**Enol**			
**1** (4-NMe_2)	N11···O(DMSO)	0.0053	0.0209	0.0012	
	N11···H(DMSO:1)	0.0111	0.0340	0.0013	-1.83
	C14···H(DMSO:2)	0.0080	0.0221	0.0009	-1.19
**5** (4-H)	C18···H(DMSO:1)	0.0025	0.0078	0.0005	-0.29
	C14···H(DMSO:2)	0.0065	0.0216	0.0010	-1.07
	N11···H(DMSO:3)	0.0055	0.0194	0.0010	-0.87
	H9···S(DMSO)	0.0052	0.0171	0.0009	-0.81
	N11···S(DMSO)	0.0046	0.0144	0.0008	
	C19···S(DMSO)	0.0067	0.0199	0.0009	
**10**(4-NO_2)	N(nitro)···O(DMSO)	0.0083	0.0363	0.0018	
	O1(nitro)···H(DMSO:1)	0.0073	0.0284	0.0015	-1.28
	O2(nitro)···H(DMSO:1)	0.0051	0.0221	0.0013	-0.95

Table S7: SAPT0/def2-TZVPD interaction energy and its components [kcal/mol] for the analyzed systems (last column, D/E, indicates the dispersion-to-electrostatic ratio); detailed description of the SAPT0 components can be found in Refs.[S3–S5]

	Electrostatics	Exchange	Induction	Dispersion	SAPT0	D/E
			**Amide**			
**1** (4-NMe_2)	-19.65	21.52	-6.85	-12.96	-17.93	0.66
**2** (4-OMe)	-20.18	21.78	-7.10	-12.88	-18.37	0.64
**3** (4-Me)	-19.90	21.85	-7.11	-12.90	-18.06	0.65
**4** (3-Me)	-19.80	21.52	-6.95	-13.33	-18.55	0.67
**5** (4-H)	-20.10	21.98	-7.23	-12.81	-18.16	0.64
**6** (4-F)	-20.72	22.17	-7.44	-12.72	-18.70	0.61
**7** (4-Cl)	-20.81	22.26	-7.53	-12.74	-18.83	0.61
**8** (4-Br)	-20.80	22.29	-7.53	-12.82	-18.86	0.62
**9** (4-CF_3)	-20.98	22.40	-7.67	-12.78	-19.02	0.61
**10** (4-NO_2)	-21.47	22.69	-7.97	-12.68	-19.44	0.59
			**Enamine**			
**1** (4-NMe_2)	-14.29	11.82	-4.20	-7.76	-14.43	0.54
**2** (4-OMe)	-17.49	14.82	-5.23	-8.97	-16.87	0.51
**3** (4-Me)	-17.55	14.88	-5.25	-8.99	-16.91	0.51
**4** (3-Me)	-14.61	11.89	-4.22	-7.82	-14.76	0.54
**5** (4-H)	-17.55	14.81	-5.23	-8.96	-16.93	0.51
**6** (4-F)	-14.70	11.79	-4.17	-7.73	-14.81	0.53
**7** (4-Cl)	-14.77	11.76	-4.18	-7.72	-14.91	0.52
**8** (4-Br)	-14.90	11.90	-4.21	-7.78	-14.98	0.52
**9** (4-CF_3)	-15.09	11.99	-4.24	-7.80	-15.14	0.52
**10** (4-NO_2)	-15.31	12.01	-4.26	-7.78	-15.35	0.51
			**Enol**			
**1** (4-NMe_2)	-4.99	10.68	-2.33	-12.11	-8.75	2.43
**2** (4-OMe)	-7.77	8.42	-2.15	-5.53	-7.03	0.71
**3** (4-Me)	-5.25	8.72	-1.52	-10.05	-8.10	1.91
**4** (3-Me)	-5.47	9.35	-1.60	-11.18	-8.91	2.04
**5** (4-H)	-4.24	8.86	-1.57	-10.48	-7.44	2.47
**6** (4-F)	-3.58	8.46	-1.87	-10.74	-7.73	3.00
**7** (4-Cl)	-3.67	8.43	-1.86	-10.69	-7.79	2.91
**8** (4-Br)	-4.38	8.22	-1.80	-10.41	-8.37	2.38
**9** (4-CF_3)	-6.49	7.20	-1.88	-5.63	-6.80	0.87
**10** (4-NO_2)	-6.57	6.46	-1.84	-6.52	-8.47	0.99

References

(S1) Cremer, D.; Kraka, E. A description of the chemical bond in terms of local properties of electron density and energy. *Croat. Chim. Acta* **1984**, *57*, 1259–1281.

(S2) Espinosa, E.; Molins, E.; Lecomte, C. Hydrogen bond strengths revealed by topological analyses of experimentally observed electron densities. *Chem. Phys. Lett.* **1998**, *285*, 170–173.

(S3) Jeziorski, B.; Moszynski, R.; Szalewicz, K. Perturbation Theory Approach to Intermolecular Potential Energy Surfaces of van der Waals Complexes. *Chem. Rev.* **1994**, *94*, 1887–1930.

(S4) Parker, T. M.; Burns, L. A.; Parrish, R. M.; Ryno, A. G.; ; Sherrill, C. D. Levels of Symmetry Adapted Perturbation Theory (SAPT). I. Efficiency and Performance for Interaction Energies. *J. Chem. Phys.* **2014**, *140*, 094106.

(S5) Hohenstein, E. G.; Sherrill, C. D. Wavefunction methods for noncovalent interactions. *WIREs Comp. Mol. Sci.* **2012**, *2*, 304–326.
